# Past, Present, and Future of Non-invasive Brain Stimulation Approaches to Treat Cognitive Impairment in Neurodegenerative Diseases: Time for a Comprehensive Critical Review

**DOI:** 10.3389/fnagi.2020.578339

**Published:** 2021-01-20

**Authors:** Clara Sanches, Chloé Stengel, Juliette Godard, Justine Mertz, Marc Teichmann, Raffaella Migliaccio, Antoni Valero-Cabré

**Affiliations:** ^1^Cerebral Dynamics, Plasticity and Rehabilitation Group, FRONTLAB Team, CNRS UMR 7225, INSERM U 1127, Institut du Cerveau, Sorbonne Universités, Paris, France; ^2^National Reference Center for Rare or Early Onset Dementias, Department of Neurology, Institute of Memory and Alzheimer’s Disease, Pitié-Salpêtrière Hospital, Assistance Publique -Hôpitaux de Paris, Paris, France; ^3^Laboratory for Cerebral Dynamics Plasticity & Rehabilitation, Boston University School of Medicine, Boston, MA, United States; ^4^Cognitive Neuroscience and Information Technology Research Program, Open University of Catalonia, Barcelona, Spain

**Keywords:** neurodegenerative diseases, cognitive decline, non-invasive brain stimulation (NIBS), Transcranial Magnetic Stimulation (TMS), transcranial Direct Current Stimulation (tDCS), brain networks, cognitive training

## Abstract

Low birth rates and increasing life expectancy experienced by developed societies have placed an unprecedented pressure on governments and the health system to deal effectively with the human, social and financial burden associated to aging-related diseases. At present, ∼24 million people worldwide suffer from cognitive neurodegenerative diseases, a prevalence that doubles every five years. Pharmacological therapies and cognitive training/rehabilitation have generated temporary hope and, occasionally, proof of mild relief. Nonetheless, these approaches are yet to demonstrate a meaningful therapeutic impact and changes in prognosis. We here review evidence gathered for nearly a decade on non-invasive brain stimulation (NIBS), a less known therapeutic strategy aiming to limit cognitive decline associated with neurodegenerative conditions. Transcranial Magnetic Stimulation and Transcranial Direct Current Stimulation, two of the most popular NIBS technologies, use electrical fields generated non-invasively in the brain to long-lastingly enhance the excitability/activity of key brain regions contributing to relevant cognitive processes. The current comprehensive critical review presents proof-of-concept evidence and meaningful cognitive outcomes of NIBS in eight of the most prevalent neurodegenerative pathologies affecting cognition: Alzheimer’s Disease, Parkinson’s Disease, Dementia with Lewy Bodies, Primary Progressive Aphasias (PPA), behavioral variant of Frontotemporal Dementia, Corticobasal Syndrome, Progressive Supranuclear Palsy, and Posterior Cortical Atrophy. We analyzed a total of 70 internationally published studies: 33 focusing on Alzheimer’s disease, 19 on PPA and 18 on the remaining neurodegenerative pathologies. The therapeutic benefit and clinical significance of NIBS remains inconclusive, in particular given the lack of a sufficient number of double-blind placebo-controlled randomized clinical trials using multiday stimulation regimes, the heterogeneity of the protocols, and adequate behavioral and neuroimaging response biomarkers, able to show lasting effects and an impact on prognosis. The field remains promising but, to make further progress, research efforts need to take in account the latest evidence of the anatomical and neurophysiological features underlying cognitive deficits in these patient populations. Moreover, as the development of *in vivo* biomarkers are ongoing, allowing for an early diagnosis of these neuro-cognitive conditions, one could consider a scenario in which NIBS treatment will be personalized and made part of a cognitive rehabilitation program, or useful as a potential adjunct to drug therapies since the earliest stages of suh diseases. Research should also integrate novel knowledge on the mechanisms and constraints guiding the impact of electrical and magnetic fields on cerebral tissues and brain activity, and incorporate the principles of information-based neurostimulation.

## Introduction

Due to low birth rates and increasing life expectancy, developed societies are facing rapid population aging. Consequently, health systems have to deal with dramatic increases of the incidence and prevalence of cognitive decline and aging-related diseases mainly due to neurodegenerative pathologies, which significantly impact daily life (Przedborski et al., 2003).

It has been estimated that about 24 million people worldwide suffer from cognitive neurodegenerative diseases (Qiu et al., 2007), and their prevalence doubles every five years (Fratiglioni et al., 2008). These pathologies are individually and socially debilitating and represent an unbearable burden for patients and their families, especially due to the lack of effective treatments to either stop or contain the clinical progression (Przedborski et al., 2003). In this context, the development of novel therapeutic approaches able to drive improvements in quality of life, and dwindle their associated clinical, social and financial burden becomes paramount.

Pharmacological strategies have been privileged during the last decade (Emre et al., 2004; Vossel and Miller, 2008; Kumar et al., 2015), however, they have been unable to consistently prove efficacy in controlled clinical trials. One recent paradigmatic example of mitigated and still inconclusive results is the Phase III trial of Aducanumab, a monoclonal antibody studied in Alzheimer’s disease (AD; Haeberlein et al., 2017). Similarly, cognitive training and rehabilitation strategies, supported in some cases by *gamified* mind training software (Legouverneur et al., 2011; Padala et al., 2012), virtual reality (Buss, 2009) or web-based rehabilitation (Tarraga et al., 2006) have provided circumstantial relief, but remain far from showing a real therapeutic impact changing prognosis. The failure of such therapy-aimed protocols is also due to the fact that the enrolled subjects in most of the trials are too advanced on the clinical, and probably histopathological, level to result in a therapeutically meaningful benefit.

Effective therapeutic approaches in neurodegeneration should be able to operate on the degenerative process itself or, alternativey, on brain plasticity to generate enduring modulations of excitability/activity in anatomical systems impacted by the disease or in spared neural networks interconnected with the former (Gutchess, 2014). In this vein, non-invasive brain stimulation (NIBS) approaches (also referred to as *electroceuticals*) based on Transcranial Magnetic Stimulation (TMS) or transcranial Direct Current Stimulation (tDCS) have been shown to enable plastic reorganization processes. On that basis, they have been extensively used for more than a decade on healthy participants to explore the modulation of various cognitive processes. Moreover, NIBS has been applied therapeutically to improve abnormal brain function in several conditions, including neuropsychiatric disorders such as depression (Haraldsson et al., 2004; Nitsche et al., 2009; Berlim et al., 2013), comprising a long-term antidepressant efficacy for drug-resistant major depression (Concerto et al., 2015), chronic pain (Antal et al., 2010), or the rehabilitation of motor function, attention and language impairments caused by stroke (Fridriksson et al., 2011; Marangolo et al., 2011; Bucur and Papagno, 2019; Xiang et al., 2019). Recently, some studies using NIBS have shown promise improving cognitive processes related to memory and language in aging (Reinhart and Nguyen, 2019) or neurodegenerative patients (Cotelli et al., 2011; Boggio et al., 2012; Doruk et al., 2014; Teichmann et al., 2016). The mechanistic approach subtending such therapeutic uses has been to long-lastingly modulate local function in key cortical locations, an effect that is spread-out via structural connectivity to other regions belonging to the same network, rebalancing abnormal activity levels between their nodes (Sale et al., 2015).

For the future, the aim in therapeutic research for NIBS will be to act as early as possible, as in any pathological condition. The research and development of *in vivo* disease biomarkers allowing for an early, sometimes pre-clinical diagnosis is a field advancing at a fast pace. Once the factors defining people at high risk for developing a given neuro-cognitive disease have been identified, these will serve to indentify which patients will be most likely to benefit from NIBS-based therapy. In this vein, the ultimate goal will be to treat before the disease has destroyed an important amount of neuronal tissue, and to apply patient-customized integrative approaches in which NIBS is associated with cognitive rehabilitation or favors the action of a pharmacological molecules.

In this review, we will first present a brief overview covering the mechanisms of the two most widely used NIBS techniques applied to neurodegenerative diseases (TMS and tDCS). We will then present the main clinical, anatomical and physiological features of seven relevant neurodegenerative diseases affecting cognition, which have been explored or treated with NIBS approaches: AD, Parkinson’s Disease (PD), Dementia with Lewy Bodies (DLB), Primary Progressive Aphasias (PPA), behavioral variant of Frontotemporal Dementia (bv-FTD), Corticobasal Syndrome (CBS), Progressive Supranuclear Palsy (PSP), and Posterior Cortical Atrophy (PCA). For each of these diseases, we will present the state of art on exploratory or therapeutic TMS/tDCS investigations aiming at modulating cognitive impairments, and discuss the consistency and relevance of such evidence. Finally, using the framework of so-called *information-based neurostimulation* (Romei et al., 2016), we will sketch out three innovative directions which could impact the field: the importance of network distributed effects, the need to integrate recent knowledge on the mechanisms guiding the impact of electrical fields on brain state and task-related activity subtended by brain oscillatory/synchrony strategies.

## Non-Invasive Brain Stimulation Therapeutic Strategies

The two most commonly employed noninvasive technologies to modulate cortical activity in neurodegeneration are TMS and tDCS.

### Transcranial Magnetic Stimulation

Transcranial Magnetic Stimulation is a focal brain stimulation technology, using brief-lasting magnetic field to painlessly convey electrical current into a brain cortical area. Such current has a sufficient intensity to trigger action potentials in the stimulated region. Developed by Anthony Barker in the mid-eighties, it was initially used to estimate pathway integrity and measure central conduction times in the cortico-spinal tract (Barker et al., 1985). Since the mid-nineties, TMS has been adopted by the cognitive neurosciences as a tool allowing to causally probe correlational links between cortical regions, their associated networks, and specific cognitive operations in healthy participants. It has also been used to probe the existence of functional brain interactions between stimulated brain regions organized in long-range networks (Pascual-Leone et al., 2000). Given its ability to modulate connectivity, TMS has also been largely used to estimate and reestablish adequate levels of local excitability in damaged brains (see Valero-Cabré et al., 2017; Polania et al., 2018 for detailed reviews).

**FIGURE 1:**
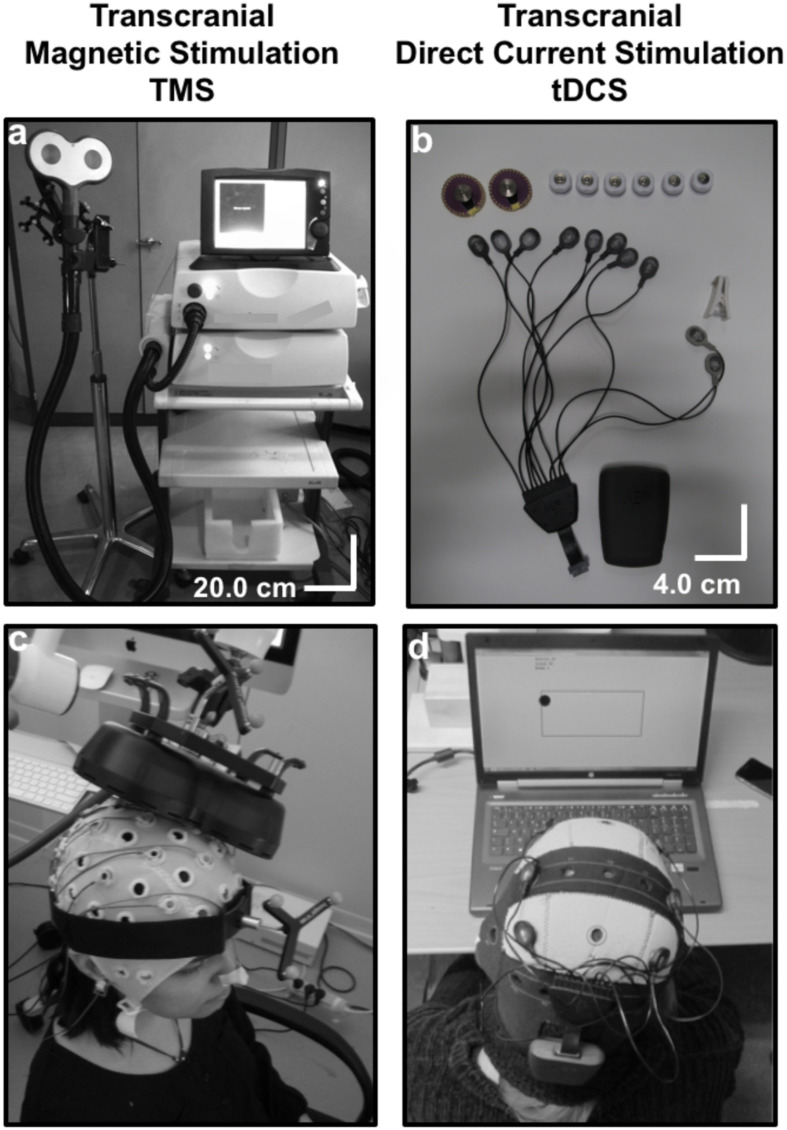
Technical equipment and procedure to use Transcranial magnetic stimulation (TMS) and Transcranial Direct Current Stimulation (tDCS): **(a)** TMS is a heavy non-portable equipment that charges current in a series of capacitors. Under the control of a bulky central unit delivers current through a coil (in the picture a “butterfly” 70 mm coil). The shape and size of the coil and pulse intensity determines the penetrability and the current density achieved on the selected cortical target. **(b)** tCS associated patterns (tDCS, tACS, and tRNS) are delivered through a portable rechargeable battery system and controlled wirelessly from a computer or portable device. Current is conveyed by wires in a montage of leads (at least two, an anode and a cathode) on specific scalp locations. The distribution of the electrical field and current density depend on current intensity, electrode size and their spatial distribution. **(c)** In TMS, a stimulation coil is placed on a subject’s head and held manually by an operator or with help from a mechanic arm or a TMS robot. Participants need to remain motionless to ensure consistent targeting, which is monitored by an MRI-based neuronavigation system. **(d)** A tDCS device is mounted directly on a lycra cap worn by a participant. In the figure, additional channels of the tDCS equipment record EEG activity. tDCS can be controlled wirelessly, and at difference to TMS is compatible with head and body motion.

In order to stimulate a brain area, a TMS coil made of cooper wire windings is placed on a specific scalp area overlying a patient’s brain region of interest, previously identified by a brain Magnetic Resonance Imaging (MRI; Figures 1a,c). A magnetic field is generated by releasing current through the coil. Obeying Faraday’s laws of electromagnetic induction (d’Arsonval, 1896; Faraday, 1914), this brief pulsed field will induce an electrical current on the cortical region underlying the TMS coil. TMS effects (which progress from cortical surface to depth and have shown a distance- and time-dependent decay intensity) concentrate mainly on directly targeted cortical regions at the surface of the brain. Nonetheless, this technique has also shown an ability to influence areas that are distant, yet anatomically and functionally connected to the directly stimulated region (Paus et al., 1997, 2001a; Chouinard et al., 2003; see also Valero-Cabré et al., 2005, 2007, in animals, reviewed in Wagner et al., 2007b). In order to study the spatial and temporal extent of their effects, TMS patterns have been combined with online Positron Emission Tomography (PET; Paus et al., 1997, 2001a; Chouinard et al., 2003), functional MRI (fMRI) (Ruff et al., 2006; Bestmann et al., 2008), *online* and *offline* Electroencephalography (EEG; Ilmoniemi et al., 1997; Taylor et al., 2007; Thut et al., 2011), and *offline* Magnetoencephalography (MEG) recordings (Marshall et al., 2015).

Transcranial Magnetic Stimulation protocols can use either *single-pulse TMS* (sTMS) to localize or map cortical function representations, *double (or paired) pulse TMS* (dTMS) to study intracortical local or distant modulatory mechanisms or repetitive TMS (rTMS) patterns to lastingly modulate activity beyond the duration of stimulation (reviewed in Rossi et al., 2009; Valero-Cabré et al., 2017; Polania et al., 2018). The impact of dTMS depends essentially on the intensity of the generated field and the location of the coil and the inter-stimulus interval between the two pulses. rTMS capitalizes also on the impact of longer lasting patterns, determined by pulse temporal distribution organization (pattern frequency) and the distribution of TMS-free intervals discontinuing frequency bursts (Miranda, 2013; Polania et al., 2018 for a review).

As a recent innovation in the field, short patterns of rTMS, known as rhythmic TMS, have been used to locally entrain or influence frequency-specific rhythmic oscillations of clusters of neurons coding for the activation of specific cognitive operations across large-scale networks (Thut et al., 2011). The use of regular rhythmic patterns of pulses combined with online EEG recordings showed a progressive induction of power increases and phase alignment of local circuits at the frequency paced by the stimulator. It allows the manipulation of local and larger-scale synchrony tied to specific cognitive operations (Thut et al., 2011, 2017).

Depending on pulse frequency and following long-term potentiation (LTP) or long-term depression (LTD)-like phenomena (Paulus et al., 2013), rTMS has shown to generate, via an impact on intracortical interneurons, either a lasting excitatory effect when delivered at ∼5 Hz and above, or an inhibitory impact when used at frequencies of 1 Hz and below (Aydin-Abidin et al., 2006 in animals; Rossi et al., 2009 for a review). These effects (*off-line* TMS effects) tend to remain active for at least half of the stimulation time, and their duration depends strongly on the temporal organization of the TMS pulses, the targeted cortical site, and also the behavioral measure chosen to evaluate the impact (Thut and Pascual-Leone, 2010). Importantly, longer lasting effects of stimulation can be achieved by repeating stimulation periodically with an interval of less than 24 h (Valero-Cabré et al., 2008), opening novel options for therapeutic uses in neurology and psychiatry.

The relevance of TMS in research, diagnostic or therapeutic applications is based on its excellent focality, particularly for well-identified cortical targets (see Valero-Cabré et al., 2005, 2007 for some high-resolution estimations in animal metabolic studies). Nonetheless, TMS carries a risk to induce epileptic seizures, particularly when used at high stimulation frequencies on individuals who, due to their clinical condition, genetic background or ongoing pharmacological treatment are prone to seize (Rossi et al., 2009). It is also contraindicated to apply TMS to patients who carry scalp or canial and intracranial implants and eventually also cardiac pacemakers, with magnetic-paramagnetic components, which could be disabled, warmed up or moved from their body locations during the stimulation (Rossi et al., 2009).

Sham approaches are generally used in basic and clinical research to ensure that the observed effects are caused by the intended neural activity manipulation and not by other potental side, or placebo, effects. Sham conditions in TMS refer to any approach that aims at mimicking the auditory and/or somatosensory effects of active stimulation without delivering active stimulation to the brain (Duecker and Sack, 2015). The delivery of TMS pulses generates a light scalp tapping sensation and a clicking noise. These are only incompletely emulated by sham interventions, hence often precluding effective blinding of the patient and the TMS operator in clinical trials (see Robertson et al., 2003; also revised in Valero-Cabré et al., 2017).

### Transcranial Direct Current Stimulation

After having been investigated in the mid-sixties in animal models as a tool for cortical polarization (Bindman et al., 1964; Purpura and McMurtry, 1965), tDCS was rescued 15 years ago as a cheaper, safer and more portable technology to modulate brain activity than TMS. Transcranial DCS is based on passing a weak constant electrical current (1–2 mA) between an *active* (anode or cathode) and a *return* electrode placed on distant regions of the skull (Nitsche and Paulus, 2000) (Figures 1b,d). At difference to TMS, tDCS is unable to directly trigger action potentials in cortical neurons. It aims to polarize the targeted region, generating large areas of cortical polarization. By attracting charges and distributing them with a specific topography along the areas influenced by the active vs. return electrodes, it modulates membrane resting potentials, making neurons more or less prone to generate an action potential (Paulus et al., 2013; Rahman et al., 2013).

The stimulation electrode i.e., either the *anode* or the *cathode* depending on stimulation modality, is placed on the region of interest, while the return is placed over a region far from the target to avoid current shunting through the skin, favoring penetration (Bikson et al., 2012; Figure 1d). At the neuronal level, anodal stimulation shifts the resting membrane potential towards its firing threshold, rendering neural cells more likely to depolarize when receiving an action potential through presynaptic inputs (Nitsche et al., 2005; Radman et al., 2009; Rahman et al., 2013). Contrary, cathodal stimulation usually hyperpolarizes the resting membrane potential of neurons, hence decreasing their probability to trigger an action potential (Nitsche et al., 2005; Radman et al., 2009; Rahman et al., 2013).

The use of sinusoidally oscillated direct current passed between an active and a set of return electrodes has given rise to a submodality of DCS known as transcranial Alternate Current Stimulation (tACS). This recent approach has shown an ability to entrain oscillatory activity and promote frequency-dependent synchrony effects across large brain networks, favoring frequency specific synchrony (Marshall et al., 2006; Kanai et al., 2008, see Reinhart and Nguyen, 2019 for a recent application). Another variation of tDCS and tACS, consists in the use of randomly oscillated direct current known as Random Noise Stimulation (tRNS) and might have the likely ability to add “noise” into extended brain areas, precluding the buildup of specific oscillations or desynchronizing ongoing brain rhythms (Thut et al., 2017). Both tACS and tRNS are currently seldomly used as NIBS therapeutic tools but they might be called to play an importat future role in the manipulation of abnormal oscillatory patterns associated to impaired brain function.

Since current flows between relatively large electrodes separated away, tDCS has a broad spatial resolution (∼5–7 cm radius with classical two electrode montages), with wide current dispersion (Wagner et al., 2007a; Bikson et al., 2009). Nonetheless, focality can be increased reducing electrode size or implementing additional montages with an active electrode surrounded by several returns (Minhas et al., 2010; Miranda, 2013). Depending on electrode size, intensities below 0.4 mA do not induce meaningful after-effects (Nitsche and Paulus, 2000), whereas those above 3 mA, particularly passed through small electrodes can induce skin rush and tinkling sensations (Furubayashi et al., 2008). Both exploratory and therapeutic tDCS effects have been obtained with intensities between 1-2mA delivered through rectangular or round electrodes (normally between 25 and 35 cm^2^). Nonetheless, individual anatomical features such as skull shape, cortical thickness, cerebrospinal fluid volume, and cortical surface topography can influence tDCS spatial distribution patterns even more largely (Wagner et al., 2009; Datta et al., 2012; Opitz et al., 2015).

The strongest assets of tDCS compared to TMS are its low cost, an outstanding safety profile [side-effects are limited to local tinkling and/or an itching sensation under the active electrode (Iyer et al., 2005)], its portability and highly adaptable ergonomics (reviewed in Valero-Cabré et al., 2017; Polania et al., 2018). These advantages have developed tDCS applications in both hospitals and particular homes for bedridden patients, boosting the popularity of this technology in clinical applications (Elder and Taylor, 2014). Moreover, in contrast with TMS, tDCS allows a reliable sham condition, which cannot be easily identified from active stimulation (Gandiga et al., 2006). The main weakness of tDCS compared to TMS is its poor spatial resolution, which is paramount when specific brain areas must be stimulated selectively (Torres et al., 2013). Its limited focality may, however, prove beneficial when cortical targets are elusive or when a clinically effective application requires, such as it is often the case in neurodegenerative diseases, the stimulation of large cortical areas (Torres et al., 2013).

**FIGURE 2:**
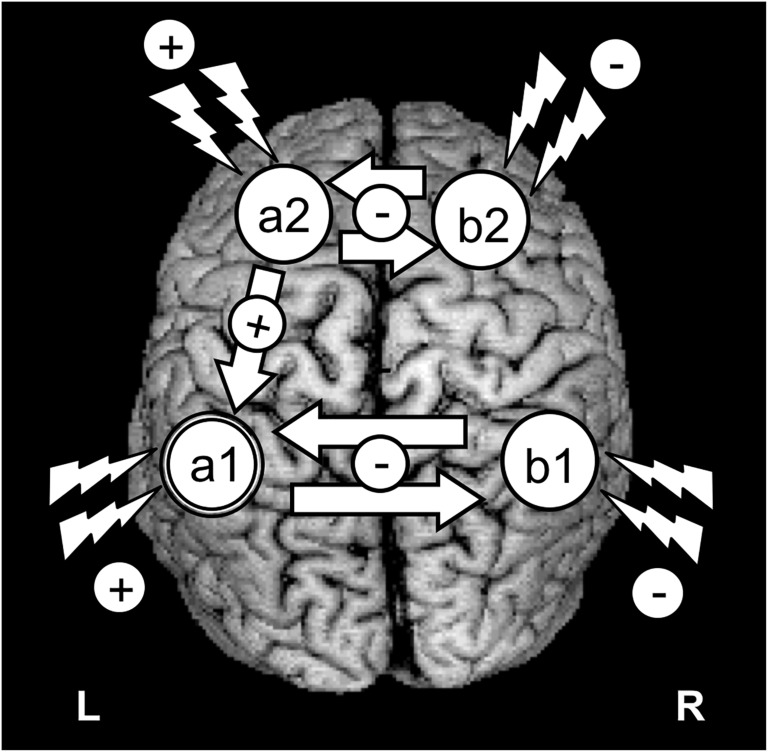
Schematics of the most common strategies to modulate the activity of a key brain region with TMS or tDCS. The figure represents an idealized scenario in which the modulation of a cognitive process depends on increasing/modulating the activity of area “a1” in the left hemisphere. Given the role of area “a1” as part of a network involving intra-hemispheric connectivity (with net excitatory effects) and inter-hemispheric connectivity (with net inhibitory effects), 4 strategies can be envisioned to achieve recovery: (1) To target directly the left “a1” area by delivering excitatory rTMS (high frequency or iTBS) or tDCS (anodal stimulation); (2) To achieve the former effect indirectly, by suppressing with inhibitory rTMS (low frequency or cTBS) or tDCS (cathodal stimulation) the modulation that a homotopic region of the right hemisphere (“b2”) exerts onto region “a1,” via transcalosal interactions; (3) Additionally, following connectivity-based principles, to activate a region of the same left hemisphere (“a2”) sustaining excitatory interactions with area “a1”; or (4) to aim at an indirect effect by suppressing the inhibition that right hemisphere area “b2” exerts on left hemisphere area “a2,” by exciting the former area, “a1.”

The use of rTMS or tDCS for improving brain function is currently developing around two main strategies: (1) to enable increases of cortical excitability within areas of interest hosting specific cognitive operations (i.e., to promote improvements in performance likely by facilitating LTP-like processes between the stimulated neurons); and/or (2) to suppress networks (likely via LTD-like processes) in damage-spared brain areas that under normal conditions interfere performance (Liebetanz et al., 2002; Luber and Lisanby, 2014). The latter approach is often achieved by reducing an output of net inhibitory interactions from a healthy area located in the contralateral hemisphere, relative to the cognitively relevant homotopic region, which releases the latter from a pathological state of excessive transcallosal inhibition (Hamilton et al., 2010; Floel, 2014; Luber and Lisanby, 2014; Figure 2). However, an alternative neurorehabilitative model, proposed by Di Pino et al. (2014) based on the study of stroke patients, suggests a bimodal balance-recovery model, by introducing and defining the concept of structural reserve. According to its tenets, the extent to which neural pathways and relay stations spared by the lesion could contribute in a given patient for a specific function to recovery (aka. the structural reserve) needs to be characterized, and guide the optimal choice of a stimulation strategy.

## Non-Invasive Stimulation in Neurodegenerative Diseases

The present literature search for publications in the field NIBS and neurodedegenerative disease affecting cognition was performed using PubMed, Google Scholar, and Web of Sciences databases. We used the following search terms: “tDCS” or “transcranial direct current stimulation” or “TMS” or “transcranial magnetic stimulation” or “NIBS” or “non-invasive brain stimulation”) AND “neurodegenerative disease” or “dementia” or “cognitive functions” or “cognition” or “Alzheimer’s disease” or “Parkinson’s disease” or “Levy body dementia” or “primary progressive aphasia” or “semantic dementia” or “frontotemporal dementia” or “posterior cortical atrophia”. Search queries were as follows: “*transcranial direct current stimulation* in *Alzheimer’s disease*,” with the terms in italics (in the example, “*transcranial direct current stimulation*” and “*Alzheimer’s disease*” being replaced each time by the other previourly mentioned keywords. The following set criteria were used to screen identified sources:

1. Articles published in English.
2. Articles that appeared in peer-reviewed journals or in conference publications.
3. Articles published until September 23, 2020 (last search date).

We excluded studies which did not present original research, did not specify statistic analysis for each specific population when mixed populations were included or did not include the analysis of NIBS impact on cognitive deficits or cognitive functions, either as thir primary or secondary outcome.

After review of all studies we extracted (i) population and sample size, (ii) stimulation type and parameters, (iii) stimulated brain regions, (iv) presence/absence of a sham condition, (v) study design vi) presence/absence of tasks during the stimulation period and vii) outcome measures and results. To rate the relative strength of the results obtained by each study and their therapeutic evidence, the Classification of Evidence Schemes- Criteria for Rating Therapeutic Studies^1^ was used. Information about the published studies is reported in Table 1.

**TABLE 1:** Comprehensive list of studies assessing the impact of TMS or tDCS on cognitive function in different neurodegenerative diseases.

References	Stimulation technology	Stimulation parameters	Stimulation targets	Active vs Sham	Study design	Online vs. Offline tasks	Outcomes	*n*	Class*
**Alzheimer’s Disease**									
Ahmed et al., 2012	rTMS + TMS-EMG recordings	Low and High frequency (1 and 20 Hz)	Right and left DLPFC consecutively	✓	1 session/day for 5 days; 3 randomized groups	No online tasks	Improvement after high rTMS in MMSE, IADL and GDS scales, maintained for 3 months and associated with a reduction of the duration of transcallosal inhibition	45	I
Bentwich et al., 2011	rTMS	High frequency (10Hz)	Broca’s and Wernicke’s areas, right and left DLPFC, right and left pSAC simultaneously	✓	1 session/day, 5 days/week for 6 weeks, followed by 2 days/week for 3 months	Online cognitive tasks/Offline evaluation	Improvement of ADAS-cog and CGIC scales after 6 weeks, maintained after the 3 months	8	IV
Boggio et al., 2012	tDCS	Anodal (2mA, 0.057mA/cm^2^, 30 min)	Right and left temporal cortex simultaneously	✓	1 session/day for 5 days; randomized sham and active sessions separated by 70 days	No online tasks	Improvement in a Visual recognition memory test, maintained for 4 weeks	15	II
Boggio et al., 2009	tDCS	Anodal (2mA, 0.057mA/cm^2^, 30 min)	Left DLPFC; left temporal cortex	✓	Single sessions; randomized and counterbalanced order	No online tasks	Improvement in visual recognition memory immediately after DLPFC and temporal cortex stimulation	10	III
Brem et al., 2013 *Published as an Abstract*	rTMS + TMS-EMG recordings	High frequency (10Hz)	Broca’s and Wernicke’s areas, right and left DLPFC, right and left parietal cortex simultaneously	✓	1 session/day, 5 days/week for 6 weeks; 2 randomized groups	Online cognitive tasks/Offline evaluation	Improvement of ADAS-cog scale within the first month after treatment. No changes in active/resting motor thresholds or amplitude of motor evoked potentials	_	IV
Brem et al., 2020	rTMS + TMS-EMG recordings	High frequency (10Hz)	Left IFG, left STG, right and left DLPFC, right and left IPL simultaneously	✓	1 session/day, 5 days/week for 6 weeks; 3 randomized groups	Online cognitive training	Improvement in the ADAS-cog immediately after and 4-6 weeks after the end of treatment. No changes in neurophysiological measures	34	II
Bystad et al., 2016a *Published as a Letter to the Editor*	tDCS	Anodal (2mA, 0.057mA/cm^2^, 30min)	Left temporal cortex	✓	2 sessions/day during a 6-day period	No online tasks	Improvement in MMSE and CVLT-II, maintained after 2 months	1	IV
Bystad et al., 2016b	tDCS	Anodal (2mA, 0.057mA/cm^2^, 30min)	Left temporal cortex	✓	6 stimulation sessions in a period of 10 days; 2 randomized groups; double-blind study	No online tasks	No effects in MMSE, CVLT-II, Trail Making Test, clock drawing	25	I
Bystad et al., 2017	tDCS	Anodal (2mA, 30min)	Left temporal cortex	✓	Daily sessions for 8 months	No online tasks	Improvement of immediate and delayed recall tests after 8 months and maintenance of other general cognitive functions	1	IV
Coppi et al., 2016 *Published as an Abstract*	rTMS	High frequency (10Hz)	Fronto-parieto-temporal lobes	✓	3 sessions/week for 4 weeks followed by 1/week for another 4 weeks; 2 randomized groups	No online tasks	Improvements in the ADAS-cog scale immediately after the 1^st^ and 2^nd^ phases of treatment	26	IV
Cotelli et al., 2014a	tDCS	Anodal (2mA, 0.08mA/cm^2^, 25min)	Left DLPFC	✓	1 session/day, 5 days/week for 2 weeks; 3 randomized groups; double-blind study	Online memory or motor tasks/Offline evaluation	No specific effects of tDCS on the Face Name Association Memory Task	36	II
Cotelli et al., 2011	rTMS	High frequency (20Hz)	Left DLPFC	✓	1 session/day, 5 days/week for 4 weeks; 2 randomized groups	No online tasks	Improvements in auditory sentence comprehension, maintained after 8 weeks	10	III
Cotelli et al., 2008	rTMS	High frequency (20Hz)	Right DLPFC; left DLPFC	✓	Single session; randomized order	Online naming tasks/Online evaluation	Improvements on picture naming during stimulation	24	III
Cotelli et al., 2006	rTMS	High frequency (20Hz)	Right DLPFC; left DLPFC	✓	Single session; randomized order	Online naming tasks/Online evaluation	Improvements in Picture Naming Task during right and left stimulation	15	III
Eliasova et al., 2014	rTMS	High frequency (10Hz)	Right IFG; Right STG	✓	Single sessions; randomized and counterbalanced order	No online tasks	Improvements in the Trail Making Test immediately after right IFG stimulation	10	IV
Ferrucci et al., 2008	tDCS	Anodal and Cathodal (1.5mA, 0.06mA/cm^2^, 15 min)	Right TPC; left TPC	✓	Single sessions; randomized and counterbalanced order; double-blind study	No online tasks	Improvements in a word recognition memory test immediately after AtDCS, decline after CtDCS	10	III
Gangemi et al., 2020	tDCS	Anodal (2mA, 2.5mA/cm^2^, 20min)	Left frontotemporal cortex	✓	Study 1: 1 daily session during 10 days/Study 2: 1 daily session in 10 consecutive days each month for 8 months	No online tasks	Study 1: improvements in the temporal orientation subscale of the MMSE/Study 2: maintenance of MMSE scores for the experimental group while significant decrease in scores for the sham group	Study 1: 26/Study 2: 18	II
Hung et al., 2017	tDCS	Anodal (1.5mA, 0.3mA/cm^2^, 20min)	Left TPC	✓	1 session/day for 10 days	Semantic feature training	No changes after stimulation in a semantic feature task	1	IV
Im et al., 2019	tDCS + FDG-PET	Anodal (2mA, 0.07mA/cm^2^, 30min)	Left DLPFC	✓	1 session/day for 6 months; randomized groups; double-blind study	No online tasks	Improvement in the MMSE and BNT scores after the intervention. Maintenance of the metabolic rate of glucose in the middle/inferior temporal gyrus while decrease in the sham group.	18	I
Khedr et al., 2014	tDCS + TMS-EMG recordings	Anodal and Cathodal (2mA, 0.083mA/cm^2^, 25 min)	Left DLPFC	✓	1 session/day for 10 days; 3 randomized groups; double-blind study	‘aq1	Improvements in MMSE after AtDCS and CtDCS associated with a reduction of P300 latency, further improvement after 1 month and after 2 months. No changes in active/resting motor thresholds	34	I
Khedr et al., 2019	tDCS	Anodal and Cathodal (2mA, 0.057mA/cm^2^, 20 min + 20 min)	Left and Right TPC sequentially	✓	1 session/day for 10 days; 2 randomized groups; double-blind study	No online tasks	Improvements immediately after the treatment in the MMSE, MoCA, clockdrawing test and Cornell Depression scale associated with changes in plasma Aβ 1-42 protein	44	I
Koch et al., 2018	TMS + EEG recordings	High frequency (20Hz)	Precuneus	✓	1 session/day for 2 weeks; double-blind study	No online tasks	Improvements in episodic memory. Changes in functional connectivity, brain oscillations and neural activity	14	II
Lee et al., 2016	rTMS	High frequency (10Hz)	Broca’s and Wernicke’s areas, right and left DLPFC, right and left pSAC simultaneously	✓	1 session/day, 5 days/week for 6 weeks; 2 randomized groups; double-blind study	Online cognitive tasks/Offline evaluation	Improvement in ADAS-cog, MMSE and CGIC scales, maintained after 6 weeks	26	II
Liu et al., 2019	tDCS	Anodal (2mA, 0.057mA/cm^2^, 20min)	DLPFC bilaterally; Temporal cortices bilaterally	✓	Single sessions; randomized and counterbalanced order; double-blind study	No online tasks	Improvement of accuracy on a 2-back task for the bitemporal stimulation	17 (mixed MCI and AD)	III
Marceglia et al., 2016	tDCS + EEG recordings	Anodal and Cathodal (1.5mA, 0.06mA/cm^2^, 15 min)	Right TPC; left TPC	✓	Single sessions; randomized order	No online tasks	Improvements in a word recognition task immediately after anodal tDCS correlated with increases of oscillation power for high-frequency bands and enhancements of temporo-parieto-occipital coherence.	7	IV
Penolazzi et al., 2014	tDCS	Anodal (2mA, 0.06mA/cm^2^, 20 min)	Left DLPFC	✓	2 cycles (active/sham) of 1 session/day for 2 weeks, separated by 2 months	Offline computerized tasks/Offline evaluation	Stability of MMSE, BNE-2 after active tDCS + CT, maintained after 3 months	1	IV
Rabey et al., 2013	rTMS	High frequency (10Hz)	Broca’s and Wernicke’s areas, right and left DLPFC, right and left pSAC simultaneously	✓	1 session/day, 5 days/week for 6 weeks followed by 2 days/week for 3 months; 2 randomized groups; double-blind study	Online cognitive tasks/Offline evaluation	Improvement in ADAS-cog and CGIC scales after 6 weeks, maintained after the 3 months	15	II
Rabey and Dobronevski, 2016	rTMS	High frequency (10Hz)	Broca’s and Wernicke’s areas, right and left DLPFC, right and left pSAC simultaneously	✓	1 session/day, 5 days/week for 6 weeks	Online cognitive tasks/Offline evaluation	Improvement in ADAS-cog and MMSE scales immediately after the treatment period	30	IV
Rutherford et al., 2015	rTMS	High frequency (20Hz)	Right and left DLPFC	✓	2 blocks of 5 sessions/week for 2 weeks; cross-over study	Online picture naming task	Improvements in a word-image association task and MoCA scale after the active treatment period, stronger for the early-stage AD group	10	III
Sabbagh et al., 2019	rTMS	High frequency (10Hz)	Broca’s and Wernicke’s areas, right and left DLPFC, right and left inferior parietal lobes (3 alternate regions/session)	✓	1 session/day, 5 days/week for 6 weeks	Online cognitive tasks/Offline evaluation	Improvement in the ADAS-Cog 6 weeks after stimulation for patients with a baseline ADAS-Cog score <30	129	II
Suemoto et al., 2014	tDCS	Anodal (2mA, 0.057mA/cm^2^, 20 min)	Left DLPFC	✓	3 session/week for 2 weeks	No online tasks	No improvements in the ADAS-cog	40	I
Wu et al., 2015	rTMS	High frequency (20Hz)	Left DLPFC	✓	5 days/week for 4 weeks	No online tasks	Improvement in the ADAS-Cog scores after the intervention	52	I
Zhao et al., 2017	rTMS	High frequency (20Hz)	Right and left parietal and right and left posterior temporal lobes	✓	1 session/day, 5 days/week for 6 weeks	Online cognitive tasks/Offline evaluation	Improvement in the ADAS-cog immediately after treatment that was maintained after 6 weeks and in AVLT, MMSE and MoCA scores after 6 weeks, only for mild but not moderate AD patients	30	III
**Primary Progressive Aphasias**									
Benussi et al., 2020	tDCS	Anodal (2mA, 0.06mA/cm^2^, 20min)	Left PFC	✓	1 session/day, 5 days/week for 2 weeks; randomized, double-blind design	No online tasks	Improvements in the MMSE, phonemic fluency, TMT-B and in the digit symbol substitution test	30	I
Cotelli et al., 2012	rTMS	High frequency (20Hz)	Right DLPFC; left DLPFC	✓	Single session; randomized and counterbalanced order	Online naming task/Online evaluation	Improvements on an action-naming task during right and left stimulations	10 (nfv-PPA)	II
Cotelli et al., 2014b	tDCS	Anodal (2mA, 0.08mA/cm^2^, 25 min)	Left DLPFC	✓	1 session/day for 2 weeks; 2 randomized groups	Online speech therapy/Offline evaluation	Improvement of speech production, maintained after 12 weeks	16 (nfv-PPA)	II
Cotelli et al., 2016	tDCS	Anodal (2mA, 0.057mA/cm^2^, 25min)	Left DLPFC	✓	1 session/day, 5 days/week for 2 weeks	Online anomia training tasks/Offline evaluation	Improvements in naming accuracy, maintained after 3 months	18 (nfv-PPA)	III
De Aguiar et al., 2020	tDCS	Anodal (2mA, 0.08mA/cm^2^, 20 min)	Left IFG	✓	1 session/day, 5 days/week for 3 weeks	Online oral naming and written spelling tasks/Offline evaluation	Improvement in letter accuracy at 2 months after intervention for trained and untrained items	30 (mixed nfv-PPA, lv-PPA and sv-PPA)	I
Fenner et al., 2019	tDCS	Anodal (2mA, 0.08mA/cm^2^, 20 min)	Left IFG	✓	10 to 14 sessions in 5 sessions per week	Online oral verb naming and written spelling tasks/Offline evaluation	Improvement in verb naming for trained and untrained items after tDCS intervention, lasting for up to 2 months	11 (mixed nfv-PPA and lv-PPA)	II
Ficek et al., 2018	tDCS + fMRI acquisitions	Anodal (2mA, 0.08mA/cm^2^, 20 min)	Left IFG	✓	2 randomized cycles (1sham/1 active) of 1 session/day, 5 days/week for 3 weeks, separated by 2 months	Online oral naming and written spelling tasks/Offline evaluation	Decrease in functional connectivity between IFG and different language related areas; correlation between functional connectivity changes and improvement in written spelling.	24 (mixed nfv-PPA, lv-PPA and sv-PPA)	III
Finocchiaro et al., 2006	rTMS	High frequency (20Hz)	Left PFC	✓	3 cycles (active/sham/active) of 1 session/day for 5 days, each new cycle started once performance returned to a base-line level	No online tasks	Improvement on verb production, maintained after 2 months for the 1^st^ cycle and after 1.5 months after the 2^nd^ cycle	1 (unspecified PPA)	IV
Gervits et al., 2016	tDCS	Anodal (1.5mA, 0.06mA/cm^2^, 20min)	Left frontotemporal region	✓	1 session/day for 10 days	Online speech production task	Improvements of speech production and grammatical comprehension, maintained after 3 months	6 (mixed nfv-PPA and lv-PPA)	IV
Harris et al., 2019	tDCS + MRS acquisitions	Anodal (2mA, 0.078mA/cm^2^, 20min)	Left IFG	✓	15 sessions; double-blind study	Online oral and written naming tasks/Offline evaluation	Improvement in language scores immediately after intervention and at 2-month follow-up; Decrease in GABA levels in the left IFG immediately after intervention and maintained after 2 months	22 (mixed nfv-PPA, lv-PPA and sv-PPA)	II
Hung et al., 2017	tDCS	Anodal (1.5mA, 0.3mA/cm^2^, 20min)	Left TPC	✓	1 session/day for 10 days	Online semantic feature training	Improvements in a semantic feature task for trained items	3 (mixed lv-PPA and sv-PPA)	IV
Manenti et al., 2015b	tDCS	Anodal and Cathodal (2mA, 0.057mA/cm^2^, 25 min)	Left (anodal) and right (cathodal) DLPFC simultaneously	✓	1 session/day, 5 days/week for 4 weeks	Offline speech therapy	Improvements in verb naming, maintained after 44 weeks	1 (nfv-PPA)	IV
McConathey et al., 2017	tDCS	Anodal (1.5mA, 0.06mA/cm^2^, 20min)	Left prefrontal region	✓	1 session/day for 10 days	Online story narration	Improvements in global language performance and semantic processing only for low baseline performers.	7 (mixed nfv-PPA and lv-PPA)	IV
Roncero et al., 2017	tDCS	Anodal (2mA, 0.057mA/cm^2^, 30min)	Left inferior parieto-temporal region	✓	10 sessions	Online picture naming	Improvements in picture naming for trained items and a mild improvement for untrained items lasting at least for 2 weeks.	10 (mixed nfv-PPA, lv-PPA and sv-PPA)	II
Roncero et al., 2019	tDCS	Anodal (2mA, 0.057mA/cm^2^, 30min)	Left parieto-temporal region; left DLPFC	✓	10 sessions over 3 weeks	Online picture naming	Improvement in picture naming for trained items after both types of stimulation, maintained after 2 weeks only for parieto-temporal region stimulation. Improvement for untrained items lasting for 2 weeks after parieto-temporal stimulation.	12 (mixed nfv-PPA, lv-PPA and sv-PPA)	II
Teichmann et al., 2016	tDCS	Anodal and Cathodal (2mA, 0.06mA/cm^2^, 20 min)	Left anterior temporal cortex; right anterior temporal cortex	✓	Single sessions; randomized and counterbalanced order; double-blind study	Online visuomotor task	Improvements in a semantic association task immediately after stimulation	12 (sv-PPA)	II
Trebbastoni et al., 2013	rTMS	High frequency (20Hz)	Left DLPFC	✓	4 randomized cycles (2 sham/2 active) of 1 session/day for 5 days in a 69 day-period	No online tasks	Improvement of both oral and written language skills immediately after the 5 days of stimulation	1 (lv-PPA)	IV
Tsapkini et al., 2014	tDCS	Anodal (1-2mA, 0.04mA/cm^2^, 20 min)	Left IFG	✓	2 randomized cycles (1sham/1 active) of 1 session/day, 5 days/week for 3 weeks, separated by 2 months	Online spelling tasks/Offline evaluation	Improvement in written spelling for untrained items, maintained after 2 months	6 (mixed nfv-PPA and lv-PPA)	IV
Tsapkini et al., 2018	tDCS	Anodal (2mA, 0.08mA/cm^2^, 20 min)	Left IFG	✓	2 randomized cycles (1sham/1 active) of 1 session/day, 5 days/week for 3 weeks, separated by 2 months	Online oral naming and written spelling tasks/Offline evaluation	Improvement in written spelling for trained and untrained items, maintained after 2 months, for the nfv and lv-PPA patients. No effects of tDCS for sv-PPA patients.	36 (mixed nfv-PPA, lv-PPA and sv-PPA)	II
Wang et al., 2013	tDCS + EEG recordings	Anodal (1.2 mA, 0.048mA/cm^2^, 20 min)	Left PPR and Broca’s area consecutively	✓	4 intercalated cycles (2 sham/2 active) of 2 sessions/day (1 for each region) for 5 days	No online tasks	Improvements in PACA subtests immediately after the 5 days of stimulation along with increases in EEG nonlinear index of approximate entropy in different brain regions.	1 (nfv-PPA)	IV
**Behavioral variant of Fronto-Temporal Dementia**									
Agarwal et al., 2016 *Published as a Letter to the Editor*	tDCS	Anodal (2mA, 0.057mA/cm^2^, 20min)	Left DLPFC	✓	1 session/day, 5 days/week for 2 weeks	No online tasks	Improvement in speech output and activities of daily living (informal evaluation) and in FRS logit score	1	IV
Antczak et al., 2018	rTMS	High frequency (10Hz)	DLPFC bilaterally	✓	1 session/day, 5 days/week for 2 weeks	No online tasks	Improvements in the MoCA total score, on the visuospatial abilities subtest and on the Stroop test after the 2 weeks of stimulation	9	IV
Benussi et al., 2020	tDCS	Anodal (2mA, 0.06mA/cm^2^, 20min)	Left PFC	✓	1 session/day, 5 days/week for 2 weeks; randomized, double-blind design	No online tasks	Improvements in TMT-A and TMT-B	25	I
Huey et al., 2007 *Published as a Letter to the Editor*	tDCS	Anodal (2mA, 0.08mA/cm^2^, 40min)	Left prefrontal area	✓	Single sessions; randomized and counterbalanced order; double-blind study	Online verbal fluency tasks/Online evaluation	No improvements in verbal fluency during stimulation	10	IV
**Parkinson’s Disease**									
Biundo et al., 2015 *Published as a Letter to the Editor*	tDCS	Anodal (2mA, non-specified current density, 20min)	Left DLPFC	✓	1 session/day, 4 days/week for 4 weeks	Online cognitive training/Offline evaluation	Decrease in the written coding test immediately after the end of the treatment; improvement in the story learning test and immediate memory index of the RBANS 3 months after stimulation	24	III
Boggio et al., 2006	tDCS	Anodal (1 and 2mA, 0.029 and 0.057mA/cm^2^, 20min)	Left DLPFC; Right motor cortex	✓	Single sessions; randomized and counterbalanced order	Online working memory task evaluation	Improvements in a working memory task during 2mA stimulation of left DLPFC	18	II
Doruk et al., 2014	tDCS	Anodal (2mA, 0.057mA/cm^2^, 20 min)	Right DLPFC; left DLPFC	✓	1 session/day, 5 days/week for 2 weeks; 3 randomized groups; double-blind study	No online tasks	Improvements in the Trail Making Test after right and left stimulation, maintained for 1 month	18	II
Lawrence et al., 2018	tDCS	Anodal (1.5mA, 0.043mA/cm^2^, 20min)	Left DLPFC	✓	1 session/week for 4 weeks; 6 randomized groups	Offline cognitive training	Improvements on executive functioning, attention/working memory and language immediately after intervention with maintenance after 2 months for executive functions and attention/working memory	42	II
Mally and Stone, 1999	rTMS	Low frequency (1Hz)	Vertex	x	2 session/day for ten days	No online tasks	Improvements in the MMSE after seven days of treatment	10	IV
Manenti et al., 2016	tDCS	Anodal (2mA, 0.06mA/cm^2^, 25 min)	DLPFC contralateral to the most affected side	✓	1 session/day, 5 days/week for 2 weeks; 2 randomized groups	Online physical therapy	Improvements in the PDCRS and verbal fluency, maintained after 3 months	20	II
Manenti et al., 2018	tDCS	Anodal (2mA, 0.06mA/cm^2^, 25min)	Left DLPFC	✓	1 session/day, 5 days/week for 2 weeks	Online cognitive training	Improvements in phonemic verbal fluency after stimulation maintained for 3 months	22	I
Pereira et al., 2013	tDCS + fMRI acquisitions	Anodal (2mA, 0.057mA/cm^2^, 20 min)	Left DLPFC; left TPC	✓	Single sessions; randomized and counterbalanced order	No online tasks	Improvements in a phonemic fluency task and enhanced functional connectivity of networks involving frontal, parietal and fusiform areas after DLPFC stimulation	16	III
**Dementia with Lewy Bodies**									
Elder et al., 2016	tDCS	Anodal (2.8mA, 0.08mA/cm^2,^ 20min)	Left DLPFC	✓	Single session	No online tasks	Improvements in an attentional task immediately after the stimulation	13	III
**Corticobasal Syndrome**									
Manenti et al., 2015a	tDCS	Anodal (2mA, 0.08mA/cm^2^,7min)	Left parietal cortex; right parietal cortex	✓	Single sessions; randomized order; double-blind study	Online naming tasks/Online evaluation	Shortening of naming latency for actions during left parietal cortex stimulation	17	III
**Progressive Supranuclear Palsy**									
Alexoudi et al., 2019	tDCS	Anodal (2mA, 0.057mA/cm^2^, 30min)	Motor and Pre-motor cortex	x	1 session/day, 5 days/week for 2 weeks	No online tasks	Increase in the AVLT test immediately after 10 stimulation sessions and lasting for 1 month after treatment. Increase in phonemic fluency, MMSE and Symbol Coding-WAIS-III immediately after stimulation	8	IV
Madden et al., 2019	tDCS	Anodal (1.5mA, 0.06mA/cm^2,^ 20min)	Left DLPFC	✓	Two sessions in the same day; one day for sham and one day for active conditions	Online language tasks	Improvements in phonemic fluency and action naming during stimulation	1	IV
Valero-Cabré et al., 2019	tDCS	Anodal and Cathodal (2mA, 0.06mA/cm^2^, 20 min)	Left DLPFC; right DLPFC	✓	Single sessions; randomized and counterbalanced order; double-blind study	Online visuomotor task	Improvements in a category judgment task immediately after cathodal stimulation and in a letter fluency task after anodal stimulation	12	II
**Posterior Cortical Atrophy**									
Gramegna et al., 2018	tDCS + fMRI acquisitions	2mA, 0.057mA/cm^2^, 20min	Left DLPFC	x	2 cycles of 1 session/day, 5 days/week for 4 weeks	No online tasks	Improvement in immediate visual memory, visual gestaltic perception, visual attention, and visuo-spatial short-term memory after the 2 cycles. Increased BOLD signal bilaterally in the DLPFC, increased deactivation of DMN.	1	IV

*Neurodegenerative pathologies: lv-PPA, logopenic variant of PPA; nfv-PPA, non-fluent variant of PPA; sv-PPA, semantic variant of PPA; Brain regions: DLPFC, dorsolateral prefrontal cortex; IFG, inferior frontal gyrus; IPL, inferior parietal lobe; pSAC, parietal somatosensory associated cortex; PFC, prefrontal cortex; PPR, posterior perisylvian region; TPC, temporoparietal cortex; STG, superior temporal gyrus Scales: ADAS-cog, Alzheimer’s Disease Assessment Scale-cognitive subscale; AVLT, Auditory Verbal Learning Test; CGIC, Clinical Global Impression of Change; CVLT-II, California Verbal Learning Test; FRS, Fronto-Temporal Dementia Rating Scale; GDS, Geriatric Depression Scale; IADL, Instrumental Activities of Daily Living; MMSE, Mini-Mental State Examination; MoCA, Montreal Cognitive Assessment; PDCRT, Parkinson’s Disease Cognitive Rating Scale; PACA, Psycholinguistic Assessment in Chinese Aphasia; RBANS, Repeatable Battery of Neuropsychological Status; TMT, Trail Making Test; WAIS-III, Wechsler Adult intelligence.*

*^∗^Classification of Evidence Scheme – Class I. A randomized, controlled clinical trial of the intervention of interest with masked or objective outcome assessment, in a representative population. Relevant baseline characteristics are presented and substantially equivalent among treatment groups or there is appropriate statistical adjustment for differences. The following are also required: (a) Concealed allocation, (b) Primary outcome clearly defined, (c) Exclusion/inclusion criteria clearly defined, and (d) Adequate accounting for dropouts (with at least 80% of enrolled subjects completing the study) and crossovers (with an intent-to-treat analysis if necessary) with numbers sufficiently low to have minimal potential for bias. Class II. A randomized controlled clinical trial of the intervention of interest in a representative population with masked or objective outcome assessment that lacks one or two criteria (a)–(d) above or a prospective matched cohort study with masked or objective outcome assessment in a representative population that meets (b)–(d) above. Relevant baseline characteristics are presented and substantially equivalent among treatment groups or there is appropriate statistical adjustment for differences. Class III. All other controlled trials (including well-defined external, natural history controls) in a representative population, where the outcome is independently assessed, or independently derived by objective outcome measurement. Class IV. Studies not meeting Class I, II, or III criteria including case reports, case series, consensus or expert opinion.*

In the following subsections, we will briefly present the most frequent neurodegenerative diseases affecting cognition which have been the object of exploratory or therapeutic NIBS, and we will provide a synthesis of the results/effects of NIBS in these diseases. It is important to acknowledge that each of the neurodegenerative diseases covered by this review could be impacted by cerebrovascular damage, a pathological process which operates as a “disease-modifier,” impacting the evolution of the neurodegenerative disease (Bordet et al., 2017), that may influence the choice of the therapeutic strategy to be adopted on each case and scenario (Vinciguerra et al., 2020). In spite of its fundamental importance, the vastness of the subject prevents us from discussing further the role of cerebro-vascular disease in neurodegeneration and its implications for NIBS interventions, which will certainly deserve a review of its own.

### Alzheimer’s Disease

#### Clinical Features

**FIGURE 3:**
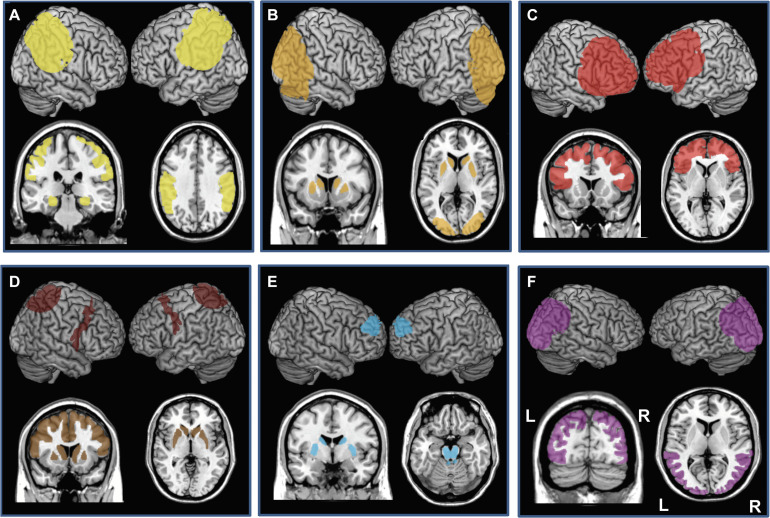
Cortical and subcortical regions affected by neurodegenerative damage in patients with **(A)** Alzheimer’s disease (AD, bilateral damage in the medial temporal lobe, hippocampus, and parietal lobe), **(B)** Dementia with Lewy Bodies (DLB, bilateral caudate and putamen, bilateral occipital and occipito-lateral cortex), **(C)** behavioral variant of Frontotemporal dementia (bv-FTD, Bilateral prefrontal cortex), **(D)** Cortico-basal syndrome (CBS, bilateral caudate and putamen, areas of prefrontal lobe, often asymmetric), **(E)** Progressive Supranuclear Palsy (PSP, bilateral caudate, putamen, midbrain and pons and bilateral circumscribed regions of the prefrontal cortex), and **(F)** Posterior Cortical atrophy (PCA, bilateral occipital, occipito-lateral and posterior parietal cortex, often right-predominant). Each panel presents left and the right hemisphere and a coronal and axial section of a standard brain.

With prevalence levels estimated around 3.9% after 60 years of age (Ferri et al., 2006) AD is the most common neurodegenerative disease affecting cognition (Hirtz et al., 2007). Typical late-onset AD patients present with memory deficits, usually associated with other cognitive or behavioral changes, leading to progressive decline impacting daily activities (Dubois et al., 2010, 2014; McKhann et al., 2011). Early and atypical forms of AD are less frequent and characterized by dysfunction in language (logopenic variant of AD), visuospatial abilities (posterior variant of AD) or executive processing (frontal variant of AD) with a relative preservation of memory (Migliaccio et al., 2009; Dubois et al., 2010, 2014). In typical late-onset AD, brain damage initially affects hippocampal neurons. Neural degeneration then extends progressively to the entire temporal lobe and to all other neocortical association areas (Scheff et al., 2007; Dubois et al., 2010; Figure 3A).

#### TMS Studies

Evidence supporting brain plasticity in individuals at risk for developing AD has steered the evaluation of NIBS (TMS and tDCS) aiming to promote plasticity on specific neural systems in AD populations (Belleville et al., 2011; Motta et al., 2018). Small cohort studies addressing the effects of TMS/tDCS on cognitive deficits in AD have shown promising benefits. High frequency rTMS over the right (Cotelli et al., 2006, 2008; Ahmed et al., 2012; Rutherford et al., 2015) and left dorsolateral prefrontal cortex (DLPFC; Cotelli et al., 2006, 2008, 2011; Ahmed et al., 2012; Rutherford et al., 2015; Wu et al., 2015) combined (Cotelli et al., 2006, 2008; Rutherford et al., 2015) or not (Cotelli et al., 2011; Ahmed et al., 2012; Wu et al., 2015) with online (during stimulation) cognitive tasks have showed beneficial effects in picture naming, auditory sentence comprehension and in the scores of the Mini-Mental State Examination (MMSE; Folstein et al., 1975), the Instrumental Activities of Daily Living (IADL; Lawton and Brody, 1988), the Geriatric Depression Scale (GDS; Yesavage et al., 1983), and the cognitive subscale of the Alzheimer’s Disease Assessment Scale (ADAS-cog scale; Rosen et al., 1984; Coppi et al., 2016). Moreover, improvements observed for the MMSE, GDS, and IADL scores (Ahmed et al., 2012) and for auditory comprehension (Cotelli et al., 2011) lasted for at least 2 months. When applied to the fronto-parietal-temporal lobes, high frequency rTMS induced improvement in the ADAS-cog scale, whereas the same pattern over the right inferior frontal gyrus (IFG) improved visual attention (Eliasova et al., 2014). A series of seven studies combining high frequency rTMS over six different brain regions combined with cognitive training during stimulation customized to activate the contributions of these regions, showed 4–18 weeks thereafter, improvement in the ADAS-cog, the Clinical Global Impression of Change score (Schneider et al., 1997; Brem et al., 2020) and the MMSE (Bentwich et al., 2011; Brem et al., 2013; Rabey et al., 2013; Lee et al., 2016; Rabey and Dobronevski, 2016; Sabbagh et al., 2019). Finally, a recent study also applying high frequency rTMS over the left and right parietal and posterior temporal lobes combined with online cognitive tasks, showed improvements that lasted for at least 6 weeks in the ADAS-cog scale, in MMSE scores, in the Montreal Cognitive Assessment (MoCA) and in an auditory verbal learning test (Zhao et al., 2017). A recent study applied high frequency rTMS over the precuneus in patients with prodromal AD and found a selective improvement in episodic memory but not in other cognitive functions (Koch et al., 2018). Importantly, the study from Brem et al. (2020) used TMS combined with electromyography (EMG) at baseline and following stimulation and showed that TMS-induced plasticity at baseline was predictive of changes of cognitive performance measured after the intervention.

#### tDCS Studies

Anodal stimulation delivered over right (Boggio et al., 2012) and left temporal cortices (Boggio et al., 2009, 2012; Bystad et al., 2016b) or over the left DLPFC (Boggio et al., 2009; Khedr et al., 2014; Im et al., 2019) and cathodal stimulation delivered over the left DLPFC (Khedr et al., 2014) improved visual recognition memory, verbal learning and MMSE scores, for at least 1 (Boggio et al., 2012) or even 2 months (Khedr et al., 2014; Bystad et al., 2016b) and in MMSE and naming scores scores after a 6-month intervention (Im et al., 2019). However, in another study, Bystad et al. (2016a) targeted the left temporal cortex with anodal tDCS and reported this time no effects on the verbal learning, visual attention or spatial organization subscores of the MMSE. Bilateral anodal stimulation over temporo-parietal regions (Ferrucci et al., 2008; Marceglia et al., 2016) and bilateral anodal stimulation over the temporal cortices (Liu et al., 2019) induced improvements in word recognition memory (Ferrucci et al., 2008; Marceglia et al., 2016) and in a 2-back task (Liu et al., 2019). In contrast, bilateral cathodal tDCSover the temporo-parietal regions entrained a decline in word recognition (Ferrucci et al., 2008). Anodal stimulation over the left temporo-parietal region immediately followed by cathodal stimulation over the right homologue region was able to improve scores on the MMSE and MoCa scales and on the clock drawing test (Khedr et al., 2019). One study applied anodal stimulation over the left frontotemporal cortex during ten days and, in a following study, during 8 months with 10 days of stimulation per month and showed that patients that underwent stimulation slowed down their cognitive decline when compared to a sham stimulation group (Gangemi et al., 2020). Two studies combined anodal tDCS over the left DLPFC with cognitive training during (Cotelli et al., 2014a) or immediately following stimulation (Penolazzi et al., 2014) and failed to reveal specific improvements in a face-to-name association memory task (Cotelli et al., 2014a), but promoted a 3-month stability of neuropsychological evaluation scores (Penolazzi et al., 2014). Only one study, that applied 3 weekly sessions of anodal tDCS over the left DLPCF, reported failure to improve cognitive functioning, attention and recognition abilities, measured by the ADAS-cog scale 2 weeks after the end of stimulation (Suemoto et al., 2014). Similarly, a single case report failed to show improvements in a semantic task after 10 days of anodal tDCS over the left temporo-parietal region (Hung et al., 2017). The only study (a single case report) by Bystad et al. (2017) that delivered daily anodal tDCS for a very long period reported no further decline in cognitive function after 8 months of daily tDCS sessions, as measured by the Repeatable Battery for the Assessment of Neuropsychological Status (RBANS; Duff et al., 2008) and an improvement in delayed and immediate recall tasks.

Interestingly, seven TMS/tDCS studies in AD associated neurophysiological biomarkers for stimulation impact (EMG responses to TMS or EEG recordings) to cognitive assessments with diverse outcomes. Ahmed et al. (2012) evaluated the duration of transcallosal inhibition (measured with paired pulse TMS stimulation) prior and following a multi-session treatment regime. Improvements of the MMSE, IADL, and GDS scores outlasting high frequency rTMS and associated with a reduction of transcallosal inhibition were observed. Regarding measures assessing M1 excitability, high frequency rTMS failed to modify active/resting motor thresholds or the amplitude of motor evoked potentials (Brem et al., 2013). Similarly, neither anodal nor cathodal tDCS applied over the left DLPFC modulated active/resting M1 motor thresholds in the left and right hemispheres (Khedr et al., 2014). However, associated improvements in MMSE scores with a reduction of the event-related potential P300 latency, a biomarker of AD (Parra et al., 2012) reflecting dysfunctional attention and memory, were reported. Koch et al. (2018) combined TMS with EEG recordings and found, after TMS, an increase of neuronal activity in the precuneus, an enhancement of brain oscillations in the beta band and also functional connectivity alterations between the precuneus and medial frontal areas. Marceglia et al. (2016) performed EEG measures prior and following bilateral anodal tDCS over the temporo-parietal regions, and reported local increases of oscillation power for high-frequency bands and enhancements of temporo-parieto-occipital coherence, scaling with improvements in a word recognition task. Abnormalities in both of these measures observed in AD patients have been associated to functional disconnections of cortical areas, the loss of cortical neurons, axonal dysfunction and cholinergic deficits (Wang et al., 2014; Marceglia et al., 2016). Finally, Gangemi et al. (2020) acquired EEG recodings prior and after tDCS over the left frontotemporal cotex to analyze activity and peak EEG frequency and found that patients that underwent active tDCS during 8 months showed no alterations on alpha, beta, or theta frequency bands while patients in the sham group showed a decrease in the alpha and beta bands. Only one study (Khedr et al., 2019) measured the effect of tDCS in neurodegenerative serum biokarkers and found that patients in the active tDCS group had an increase in the levels of plasma Aβ 1-42 protein, which was associated with the increase in cognitive measures. Only one study acquired neuroimaging measures before and after stimulation intervention (Im et al., 2019). These authors acquired FDG-PET before a protocol of daily tDCS over the left DLPFC during 6 months and once after this period. Across the time, they found equal levels of glucose metabolic rate in the middle/inferior temporal gyrus on the group receiving active tDCS, while a decrease was reported for the group receiving sham tDCS (Im et al., 2019). Finally, Im et al. (2019) produced computational models based on the MRIs of two older adults of Asian ethnicity, similar to the population included in their study. The authors showed that the montage used, with the anode placed over the left DLPFC and the cathode over the right DLPFC, entrained a current distribution affecting the frontal cortex, with peak magnitudes within a range previously reported for adults (Im et al., 2019).

Some of the above-mentioned studies also revealed stimulation effects in AD were dependent on cognitive impairment levels (hence indirectly, the clinical stage of the disease). They suggested efficacy of rTMS/tDCS within a limited window of clinical severity, with high clinical response in mild to intermediate rather than severe levels of impairment. For example, whereashigh frequency rTMS improved significantly MMSE and IADL scores in patients with mild to moderate AD, identically treated patients with severe AD did not respond to stimulation (Ahmed et al., 2012). Severity dependent outcomes were also observed in another study in which only patients with mild but not moderate AD responded to rTMS stimulation and displayed significant improvements in different cognitive scales (Zhao et al., 2017). In the same vein, two other studies also showed greater improvement in the ADAS-cog subscale (Coppi et al., 2016), the MMSE and a word-image association task (Rutherford et al., 2015) after a high frequency rTMS treatment in patients with less severe cognitive impairment at baseline. Finally, the study from Sabbagh et al. (2019), involving a large cohort of patients (*n* = 129), showed stronger improvements in mild AD patients (ADAS-Cog < 30) compared to more severely affected patients (Adas-Cog > 30). However, as the authors note, only 15% of the whole cohort belonged to the more severily impacted group and so these results should be taken carefully (Sabbagh et al., 2019).

#### Summary

Alzheimer’s disease is the neurodegenerative disase in which NIBS has been most widely evaluated, with a total of 31 published studies (4 single case reports, 2 studies with less than 10 patients, 17 studies between 10 and 30 patients, 7 studies with more than 30 patients, and 1 study not reporting the number of participants) (Table 1). Five reports recorded TMS-EMG based measures of excitability (Ahmed et al., 2012; Brem et al., 2013; Khedr et al., 2014) or EEG signatures to evaluate tDCS impact and response to treatment (Khedr et al., 2014; Marceglia et al., 2016; Koch et al., 2018). However, from these five studies, only two correlated neurophysiological measures with behavioral outcomes (Marceglia et al., 2016; Koch et al., 2018), and only a single study reported a significant correlation between these types of measures (Marceglia et al., 2016). Only one study (Khedr et al., 2019) recorded measures of serum biomarkers (plasma Aβ 1-42 protein levels) and associated these measures with cognitive outcomes. Lastly, one single study acquired neuroimaging measures before and after stimulation intervention (Im et al., 2019) to verify the impact of stimulation on neuroplasticity phenomena, but without associating such impact with behavioral measures. Four meta-analyses including 5–12 studies, evaluated the effectiveness of rTMS in cognitive impairment mostly using high frequency rTMS (Dong et al., 2018; Lin et al., 2019; Chou et al., 2020; Wang et al., 2020). All meta-analyses concluded that rTMS, compared to sham rTMS, led to significant effect-sizes, hence to statistically significant improvement in cognition, as measured with MMSE and ADAS-Cog scales (Dong et al., 2018; Lin et al., 2019; Chou et al., 2020; Wang et al., 2020). Moreover, two of thse meta-analyses performed subgroup analysis and concluded that the effects of rTMS applied to multile brain targets was greater than when applied to a single target (Lin et al., 2019; Wang et al., 2020) and that the application of more than five stimulation sessions (Liu et al., 2019) and more than 10 sessions (Wang et al., 2020) was more efficient than a lesser number of sessions. Finally, Wang et al. (2020) concluded that rTMS combined with cognitive training produced greater cognitive improvement.

Considering evidence collected for the last 10 years, high frequency rTMS and anodal tDCS delivered for at least 2 weeks have the potential to improve cognitive function in patients with AD, maximizing performance and containing the progression of cognitive decline. However, no solid evidence supports the ability of these approaches to tackle the physiopathological basis of this condition and eventually stop its course. The rapid progression combined with the broad distribution of cortical damage in AD poses a difficult scenario for these techniques. NIBS might prove either too focal (TMS) or not sufficiently intense (tDCS), and difficult to combine actively with rehabilitation, given the poor level of compliance of these clinical populations. Awaiting additional studies particularly at prodromal or early AD stages, the field has focused towards testing NIBS approaches in early onset and more focal forms of neurodegenerative diseases, in which they might prove more successful.

### Parkinson’s Disease

#### Clinical Features

Parkinson’s disease shows a prevalence among individuals equal or older than 65 years of ∼1.5% (Pringsheim et al., 2014). PD with symptoms of dementia (PDD) affects 75–90% of PD patients diagnosed for 10 years or longer (Jankovic, 2008; Aarsland and Kurz, 2010) including cognitive impairment in different domains (Dubois et al., 2007). Patients may show deficits of memory retrieval, visuoconstructive abilities, fluctuations in attention, impaired executive functions (Muslimović et al., 2005; Dubois et al., 2007) and language disabilities (Azuma et al., 2003; Muslimović et al., 2005). PD mainly affects dopaminergic neurons of the midbrain’s *substantia nigra* (*pars compacta)* (Braak et al., 2003; Berg et al., 2014). Additionally, PDD involves a disruption of fronto-striatal dopamine networks, which has been found correlated with deficits of executive function (e.g., Lewis et al., 2003).

#### TMS and tDCS Studies

The large majority of rTMS studies in PD patients have focused on treating motor disabilities. Pascual-Leone et al. (1994) carried out pioneering work showing motor improvements (tremor, rigidity, walking) in patients with PD using subthreshold low frequecy rTMS on the motor cortex. Over many years, studies in this area have supported the ability of rTMS to induce adaptive motor outcomes (Brys et al., 2016) and therapeutic benefit.

Despite a focus on motor symptoms, a growing body of evidence has shown the benefit of NIBS to treat cognitive dysfunction in PD. Mally and Stone (1999) applied low frequency rTMS over the scalp vertex of patients with PD two times a day for ten days and observed that, after 7 days of treatment, patients showed a significant improvement of MMSE scores. Manenti et al. (2016) combined physical therapy with anodal tDCS over the DLPFC contralateral to the most affected hemibody (with regards to motor performance) and showed that both motor impairments and depression symptoms improved after active and sham tDCS. Nonetheless, lasting improvements for up to three-months in Parkinson’s Disease Cognitive Rating Scale (PDCR) scores and verbal fluency were observed exclusively for the active tDCS group (Manenti et al., 2016). A recent study from Manenti et al. (2018) has shown improvement in phonemic verbal fluency after 2 weeks of daily anodal tDCS over the left DLPFC combined with online cognitive training. Importantly these effects were still present at the end of a 3-month follow-up evaluation.

Five other independent tDCS studies tested the impact of: (a) 10 consecutive sessions of anodal stimulation over the right and the left DLPFC (Doruk et al., 2014); (b) 4 weeks of 4 days/week regime of anodal stimulation over the left DLPFC coupled with cognitive training during stimulation (Biundo et al., 2015); (c) a single session per week for 4 weeks of anodal stimulation over the left DLPFC combined with cognitive training 3 times per week out of the period of stimulation (Lawrence et al., 2018); (d) A single session of anodal tDCS over the left DLPFC vs. the right motor cortex coupled to a working memory task (Boggio et al., 2006); and (e) a single anodal stimulation session over the left DLPFC and left temporo-parietal region (Pereira et al., 2013). Taken together, these studies showed that left DLPFC stimulation improved visual attention, phonemic fluency, working memory, executive functions and language/semantic abilities (Boggio et al., 2006; Pereira et al., 2013; Doruk et al., 2014; Biundo et al., 2015; Lawrence et al., 2018), whereas right DLPFC tDCS also ameliorated visual attention for at least a month (Doruk et al., 2014). One of these studies showed that, whereas no tDCS-driven effects were observed immediately after tDCS sessions, there was a significant improvement in the story learning test and the immediate memory index of the Repeatable Battery Assessment of Neuropsychological Status (RBANS; Randolph et al., 1993) 3 months after the end of the stimulation (Biundo et al., 2015).

Two additional tDCS studies in PD patients reported working memory improvements lasting for at least one month (Doruk et al., 2014) or 2 months (Lawrence et al., 2018). Importantly, in one of these reports, patients underwent a verbal fluency task during fMRI evaluation, and showed, following anodal tDCS over the left DLPFC, enhanced functional connectivity of networks involving frontal, parietal and fusiform areas (Pereira et al., 2013).

#### Summary

The only study that applied rTMS and measured its impact on cognitive outcomes suggests that this technique might impact cognitive abilities in PD. The outcomes of the seven studies that have applied tDCS to improve cognition in PD indicate that tDCS may induce changes in working memory, attention and verbal fluency, whereasthe most relevant effects appear to be generated with left DLPFC stimulation. All of these studies showed positive outcomes, a result that should encourage further research in this condition if possible combined with neuroimaging or physiological measures.

### Dementia With Lewy Bodies

#### Clinical Features

Dementia with Lewy Bodies’s prevalence is estimated in ∼0.7% of the population over 65 years of age (Kosaka, 1990). DLB patients present with fluctuating cognition with pronounced variations in attention and alertness, recurrent visual hallucinations and atypical “parkinsonism” (McKeith et al., 2005). Other areas of cognitive deficit include memory impairments, deficits in verbal fluency, executive and visuospatial functions (Salmon et al., 1996). As for PD, DLB is associated with degeneration of the *substantia nigra* and brainstem nuclei combined this time with cortical and limbic system damage (Kosaka, 1990; Figure 3B).

#### TMS and tDCS Studies

Non-invasive stimulation approaches in DLB have been only tested in a single tDCS exploratory study, lacking a sham stimulation condition. This single pre-therapeutic attempt reported improvements in attention following a session of anodal tDCS delivered over the left DLPFC (Elder et al., 2016), providing preliminary evidence of beneficial effects.

#### Summary

Additional observations using sham-controlled designs will be absolutely required to further assess the therapeutic potential of these approaches in DLB patients.

### Primary Progressive Aphasias

#### Clinical Features

**FIGURE 4:**
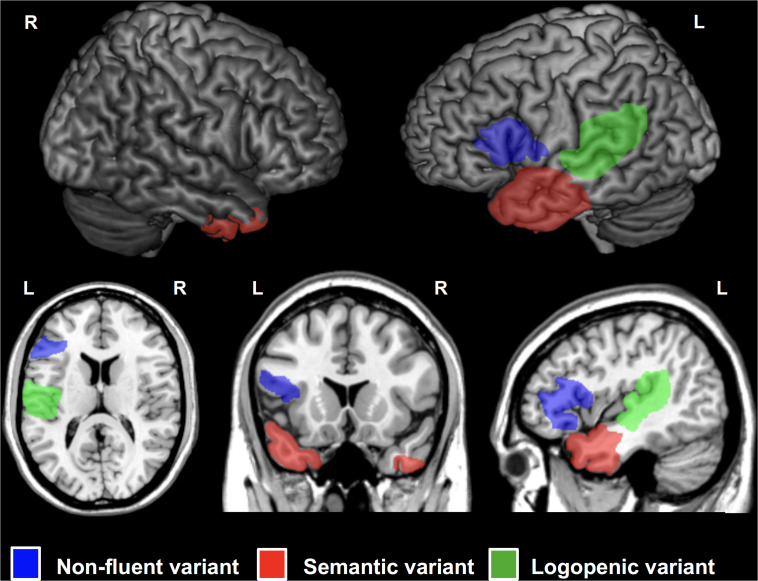
Cortical regions affected in patients with Primary Progressive Aphasia (PPA) showed on a standard brain. Specific areas for each PPA variants are indicated in different colors: semantic variant (sv-PPA, left and right anterior temporal lobes, left > right) in red, non-fluent variant (nfv-PPA, left inferior temporal gyrus around Broca’s area) in blue, and logopenic variant (lv-PPA, left posterior temporal lobes around the temporo-parietal junction) in green.

Primary Progressive Aphasia is a neurodegenerative condition generally with an onset before 65 years of age (Mesulam et al., 2014) and characterized by progressive loss of language abilities (Mesulam, 1987). Three main PPA variants have been described: *semantic* (sv-PPA), *logopenic* (lv-PPA), and *nonfluent/agrammatic* PPA (nfv-PPA). Sv-PPA is linked to damage to the anterior tempoal lobes (ATL) with a left hemisphere predominance (Figure 4). It is characterized by impairments of conceptual knowledge, resulting in anomia and difficulties in single-word comprehension (Gorno-Tempini et al., 2011). Lv-PPA affects the left temporal-parietal junction (TPJ; Figure 4) and is characterized by word-finding difficulties and a impaired verbal short-term memory (Gorno-Tempini et al., 2011). Nfv-PPA is related to damage to the left inferior-posterior frontal cortex including Broca’s area (Figure 4; Gorno-Tempini et al., 2004) and is defined by syntactic failure and difficulties in phonological and phonetic encoding leading to phonemic paraphasias and, frequently, also speech apraxia (Gorno-Tempini et al., 2011).

#### TMS Studies

Neurostimulation approaches have been probed as potential treatment to contain language deficits in the three main PPA variants. A single rTMS study explored the effects of right and left DLPFC stimulation with high frequency rTMS combined with online naming tasks in patients with nfv-PPA and reported improvements of action verb naming for both cortical targets (Cotelli et al., 2012). Regarding lv-PPA, Trebbastoni et al. (2013) reported an improvement of both oral and written language skills after high frequency rTMS over the left DLPFC in a single patient. Additionally, another single case study explored the effects of high frequency rTMS over the left prefrontal cortex (PFC) in a patient with an unspecified PPA variant, reporting improvements on verb production, enduring for at least a month and a half (Finocchiaro et al., 2006).

#### tDCS Studies

**FIGURE 5:**
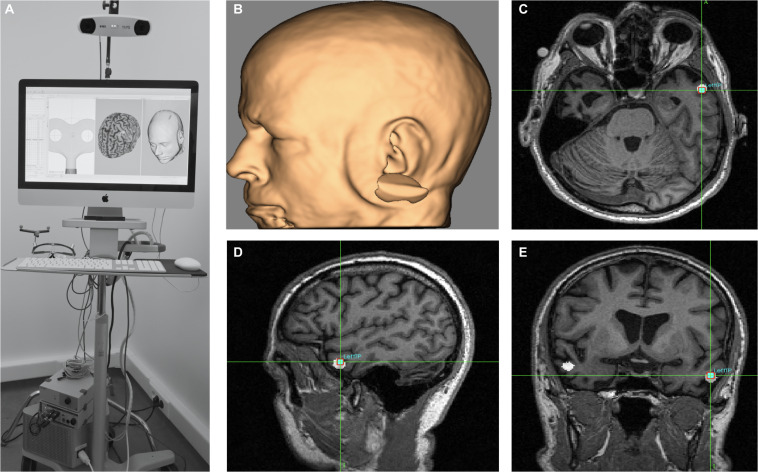
MRI-based frameless neuronavigation system used to place the stimulation devices (rTMS coil or tDCS electrodes) in optimal scalp location, overlying with the shortest-path a given cortical target. To this end, **(A)** a high resolution T1-3D MRI volume is obtained. Then cortical targets are labeled either on the basis of anatomical landmarks or by targeting MNI/Talairach coordinates (see white 5 mm radius spheres in panels c, d and e on the left and right anterior temporal lobe, ATL). **(B)** 3D reconstruction of the patient’s head surface based on his individual T13D MRI sequence. Panels **(C–E)** show axial, sagittal and coronal sections of the ATL target (see crosshairs) placed on MNI coordinates (*x* = -52, *y* = 2, *z* = -28) in a sv-PPA patient prior to an anodal tDCS treatment (as in Teichmann et al., 2016). The MRI based neuronavigation systems allow to plan pre-hoc the optimal scalp site for tDCS electrodes or site, orientation, angulation and tilting of the TMS coil.

Tsapkini et al. (2014), tested a mixed population of lv-PPA and nfv-PPA patients and reported lasting improvements in spelling for up to two months after anodal stimulation over the left IFG combined with online oral naming and written spelling tasks. Two follow-up studies, by Ficek et al. (2018) and Tsapkini et al. (2018), including a larger cohort of nfv-PPA, lv-PPA and also sv-PPA patients addressed the long-term impact of tDCS. Combining anodal stimulation over the left IFG, concomitantly with spelling and naming tasks, the authors reported improvements in spelling lasting for up to 2 months for the nfv-PPA and the lv-PPA groups. However, no beneficial effects of stimulation for the sv-PPA group were reported. More recently, this same group published three studies analyzing the results for subsets of the population involved in this same protocol, where they applied anodal tDCS to the left IFG concomitantly with spelling and naming tasks during three weeks (Fenner et al., 2019; Harris et al., 2019; De Aguiar et al., 2020). The results of these studies showed improvement in letter accuracy during written spelling both for trained and untrained items (De Aguiar et al., 2020) and in language scores (Harris et al., 2019) lasting for 2 months after treatment discontinuation for all three PPA variants. Also, improvements in verb naming for trained and untrained items, maintained for a similar period of time for a subset of nfv-PPA and lv-PPA patients (Fenner et al., 2019). Four additional studies using mixed populations of PPA variants of either lv-PPA, nfv-PPA and sv-PPA patients (Roncero et al., 2017, 2019), a combination of lv-PPA and nfv-PPA (McConathey et al., 2017) or lv-PPA and sv-PPA (Hung et al., 2017) tested respectively, the impacts of: (i) anodal left inferior parietal-temporal tDCS (Roncero et al., 2017, 2019) and anodal left DLPFC tDCS (Roncero et al., 2019) during an online picture naming task; (ii) anodal tDCS over left prefrontal regions on different language abilities (McConathey et al., 2017); and (iii) anodal tDCS over the left temporal-parietal region combined with a semantic feature training task, in which patients had to identify five semantic features of a target item, that was presented both with a picture and orally (Hung et al., 2017). These studies showed an improvement in semantic processing (Hung et al., 2017; McConathey et al., 2017) and also in picture naming for trained and untrained items (Roncero et al., 2017, 2019). The study from Roncero et al. (2019) showed that the improvement in picture naming lasted for two weeks after stimulation only when the left parietal-temporal region wasstimulated, hence not when the left DLPFC was targeted. In this study, Roncero et al. (2019) used neuronavigated tDCS (Figure 5) to precisely target the two selected cortical regions, whereas biophysical modeling of tDCS current fields served to simulate the impact of both strategies in the cortical targeted region and adjacent areas. The most recent stuy to date evaluating the effects of tDCS over the left PFC in FTD patients included a subgroup of 30 PPA patients and showed for this subgroup of patients, animprovement of phonemic verbal fluency, visual attention and task switch abilities and in MMSE scores (Benussi et al., 2020). Importantly, one study employed fMRI to analyze the effects of tDCS on functional connectivity, aiming to assess if tDCS-induced language improvements could be explained by changes in functional connectivity. Authors reported significantly lowered functional connectivity between the left IFG and other language network areas following stimulation, which correlated with tDCS-driven improvements in spelling scores (Ficek et al., 2018). De Aguiar et al. (2020) analysed baseline brain volumetric data to identify brain regions the volume of which might predict the tDCS-induced language effects. They showed that the volumes of the left angular gyrus and left posterior cingulate cortices predicted the gain in performance for trained items after tDCS whreas the volumes of the left middle frontal gyrus, left supramarginal gyrus, and right posterior cingulate cortices predicted gains for untrained items (De Aguiar et al., 2020). Finally, Harris et al. (2019) used Magnetic Resonance Spectroscopy (MRS) data and found a decrease in GABA levels in the left IFG immediately after intervention which was maintained after 2 months.

Studies in nfv-PPA patients have successfully employed anodal tDCS over the right (Manenti et al., 2015b) or left DLPFC (Cotelli et al., 2014b, 2016; Manenti et al., 2015b) combined with *offline* (not simultaneously with stimulation) (Manenti et al., 2015b, single case study) or online (during stimulation) speech therapy (Cotelli et al., 2014b, 2016). Two additional studies applied anodal tDCS to the left posterior perisylvian region and Broca’s area (Wang et al., 2013) and the left fronto-temporal region (Gervits et al., 2016). Taken together, these studies on nfv-PPA showed improvements in speech production (Cotelli et al., 2014b; Gervits et al., 2016), naming accuracy (Wang et al., 2013; Manenti et al., 2015b; Cotelli et al., 2016), grammar comprehension (Gervits et al., 2016), auditory word comprehension, oral word-reading and word-repetition (Wang et al., 2013). For some studies, post tDCS improvements lasted for a period of at least 3 months following stimulation sessions (Cotelli et al., 2014b, 2016; Manenti et al., 2015b; Gervits et al., 2016). Interestigly, the study by Wang et al. (2013) used EEG and reported changes in the nonlinear index of approximate entropy in different stimulated and non-stimulated brain regions, including left Broca’s and Wernicke’s areas, suggesting that language improvement were associated with such activations. The study from Cotelli et al. (2016) associated response outcomes with cortical grey matter density before a regime of periodical tDCS sessions over the left DLPFC and reported a positive correlation between performance improvements and grey matter density at baseline in the left fusiform, left middle temporal gyrus and right inferior temporal gyri. Additionally, the biophysical model applied in Gervits et al. (2016) showed that the current of left fonto-temporal tDCS was distributed throughout left hemisphere regions crucial for language function, hence supporting the choice of stimulation sites and electrode montages.

**FIGURE 6:**
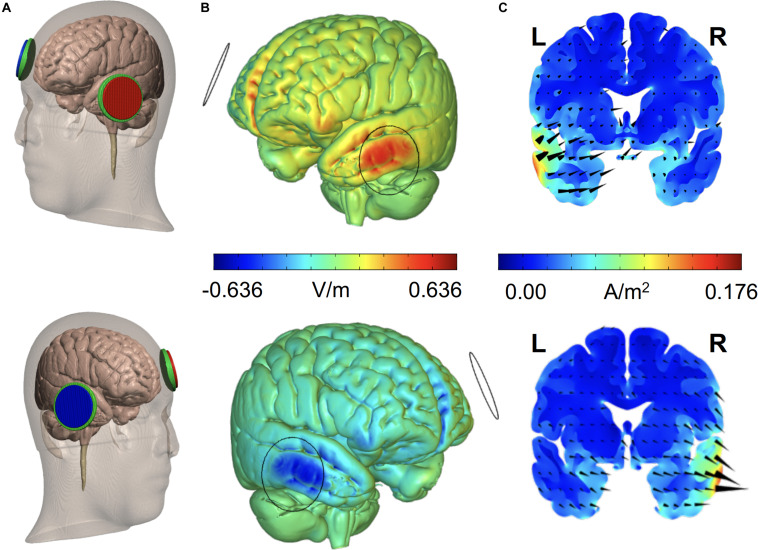
Finite Element Method (FEM) biophysical models estimate the distribution of electrical current on the brain for given sets of TMS (target site, coil type and size, and pulse intensity) or tCS (electrode location, size, montage, and intensity) parameters. Models take in consideration permittivity and volume of the tissue layers (skin, bone, epidural air space, subdural cerebro-spinal fluid (CSF), and gray and white matter) current needs to cross to reach the target. The figure shows **(A)** FEM models for two tDCS montages [**(top)** Anodal left aterior temporal lobe stimulation,: *x* = -52, *y* = 2, *z* = -28, right supraorbital cathode on AF8; **(bottom)** Cathodal right anterior temporal pole stimulation, MNI coordinates: *x* = 53, *y* = 4, *z* = -32 and left supraorbital anode on AF7) with 25 cm^2^ round electrodes at 1.59 mA intensity, employed in sv-PPA patients (Teichmann et al., 2016). For each electrode, we show **(B)** the spatial distribution of the radial electrical field (V/m) on the cortical surface, **(C)** current density (A/m^2^) and electrical flow direction on a coronal section at target. Whereas anodal tDCS increases current density and drives radial “inward” currents into the left anterior temporal lobe, cathodal stimulation in the right temporal lobe induces opposite local effects **(B,C)**. L, Left; R, Right (Adapted from Teichmann et al., 2016).

In sv-PPA, a recent double-blind cross-over pre-therapeutical trial compared the impact of single sessions of anodal or cathodal tDCS over the left and right ATL, respectively, with sham tDCS. It showed beneficial effects on a verbal semantic association paradigm after both active (anodal and cathodal) tDCS strategies (Teichmann et al., 2016). Neuronavigated tDCS was used to precisely target coordinates in the anterior third of the temporal lobe subtending semantic processing and guide electrode placement. Biophysical modeling of Direct Current fields pictured the excitatory/inhibitory impact of left anodal and right cathodal tDCS and supported stimulation sites and montages (Figure 6). Most importantly, internal semantic dissociations emphasized the intra-semantic-specificity of the effects, with higher improvements generating semantic analogies for items belonging to a “living” category, which were the most impaired at baseline in these patients (Teichmann et al., 2016). Another study correlated response outcomes with baseline performance prior to a regime of cumulative tDCS sessions and showed severity-dependent response to tDCS in sv-PPA. Nonetheless in contrast with outcomes in AD (Ahmed et al., 2012; Coppi et al., 2016), in this case, poor baseline performance was associated to better outcomes (McConathey et al., 2017).

#### Summary

Together with AD, the three PPA variants are among the neurodegenerative diseases accruing the highest number of reports with 19 studies (4 single case reports, 4 studies with less than 10 patients and 11 studies in larger cohorts). Eleven of these studies used either very small cohorts (less than 10 patients) and/or explored non-homogenous cohorts of patients mixing several PPA variants. Surprisingly, only two studies employed supportive neuroimaging (fMRI, MRS) (Ficek et al., 2018; Harris et al., 2019) and another used neurophysiological measures (EEG, MEG) (Wang et al., 2013) to verify stimulation impact or demonstrate short-term/longer-term neuroplasticity effects associated to tDCS. However, 3 studies used biophysical modeling to infer tDCS local effects and focality (Gervits et al., 2016; Teichmann et al., 2016; Roncero et al., 2019), and confirmed that electrical field spread was well distributed over the regions of interest aimed by the tDCS montage. One meta-analysis on the efficacy of language training, alone or language training during the application of tDCS, on oral and written naming deficits in PPA patients concluded that these therapies improve oral naming accuracy for trained items (Cotelli et al., 2020). Importantly, with the 7 studies included in this meta-analysis that combined language therapy with tDCS, authors concluded that only language training combined with tDCS improved naming accuracy for untrained items (Cotelli et al., 2020). Byeon (2020) also conducted a meta-analysis to evaluate the effects of tDCS on naming abilities in patients with PPA. The author analysed seven studies with mixed populations of PPA in patients that underwent tDCS concomitantly with language tasks. The effect size obtained as a result of this meta-analysis was of 0.82 (95% CI: 0.16 – 1.47), which was considered a significant large effect, suggesting that tDCS interventions significantly improved naming abilities in PPA patients (Byeon, 2020). Even if the use of rTMS and tDCS in PPA populations should be considered promising, we conclude double-blind large-scale clinical trials using therapeutic regimes including several days of stimulation in large and homogeneous PPA cohorts are needed to confirm this clinical indication.

### BehavioralVariant of Frontotemporal Dementia

#### Clinical Features

The bv-FTD is an early onset neurodegenerative disease, characterized by apathy, diminution of social convenience, impulsivity and disinhibition (Rascovsky et al., 2011). Patients also show impairments in executive functions and language production (Kramer et al., 2003; Le Ber et al., 2006; Rascovsky et al., 2011; Ranasinghe et al., 2016). Bv-FTD is characterized by atrophy of prefrontal areas such as the dorsolateral, ventromedial and orbitofrontal regions (Mackenzie et al., 2010) (Figure 3C).

#### TMS and tDCS Studies

Only 4 studies have thus far addressed the impact of NIBS on cognitive symptoms in bv-FTD. A decade old pilot study failed to detect effects on verbal fluency task of active anodal versus sham tDCS over the left prefrontal area delivered during 20 minutes (Huey et al., 2007). In contrast, a recent case report (Agarwal et al., 2016), reported improved speech production, along with ameliorations of the Fronto-Temporal Dementia Rating Scale logit scores (see Mioshi et al., 2010) and activities of daily living, following a regime of 10 consecutive days of anodal tDCS over the left DLPFC. A recent study (Antczak et al., 2018) characterized a cohort of 9 bv-FTD patients and applied high frequency rTMS to their DLPFC billateraly for 2 weeks. Authors found improvements in the MoCA total score particularly in the visuospatial subdomain as well as in the Stroop test. In a randomized, sham-controlled trial involving Frontotemporal Dementia patients that underwent tDCS stimulation over the left PFC, the subgroup of 25 bv-FTD patients showed improvements in visual attention and task switching abilities but not in Stroop, MMSE scores or phonemic verbal fluency (Benussi et al., 2020).

#### Summary

Due to the lack of significant effects of the first tDCS study in a cohort of bv-FTD patients (Huey et al., 2007), the use of purely qualitative clinical assessment and interviews in a single case report (Agarwal et al., 2016), and the lack of control group of one of the cohort studies with strong significant results, the potential of NIBS in bv-FTD patients requires further exploration before reliable conclusions on efficacy can be reached. However, the most recent study involving a large cohort of bv-FTD patients suggests that tDCS might be a promising future therapeutic strategy for these patients.

### Corticobasal Syndrome

#### Clinical Features

This syndrome is part of the Frontotemporal Lobar Degeneration spectrum (Whitwell et al., 2010; Manenti et al., 2015a). With an average onset age of 63 years (Armstrong et al., 2013), CBS patients present with strongly lateralized limb rigidity and bradykinesia. Additionally, features like dystonia, alien limb phenomenon and myoclonus have also been reported (Armstrong et al., 2013). In the cognitive domain patients often present with apraxia, aphasia, visuospatial, and executive disorders (Whitwell et al., 2010; Armstrong et al., 2013; Di Stefano et al., 2016). Brain damage varies according to the underlying neuropathology (Whitwell et al., 2010). Nonethelesss, atrophy in posteromedial frontal and peri-rolandic cortex, dorsal insula (Lee et al., 2011), right and left premotor cortex, parietal regions and the caudate and putamen are the most common features (Lee et al., 2011; Figure 3D).

#### TMS and tDCS Studies

Shehata et al. (2015) used low-frequency rTMS combined with pharmacological and rehabilitation treatment in a cohort of 26 CBS patients, stimulated over the motor cortex contralateral to the most affected side (3 times a week for 1 month every 3 months). Followed for 18 months an improvement of motor symptoms and quality of life was reported after 3 months of therapy (Shehata et al., 2015). Cognitive functions were assessed only as a secondary measure and even if patients experienced no improvements, they did not show either any sign of further cognitive deterioration over the study period (Shehata et al., 2015). Thus far, a single study has evaluated the impact of a single anodal tDCS session, compared to sham tDCS, over the left or right parietal cortex using a naming task (Manenti et al., 2015a). During left parietal cortex stimulation, patients displayed a reduction of vocal reaction times for the naming of actions (in which they were mostly impacted) but not for objects. Interestingly, the authors also reported recovery size-effects that scaled with the level of baseline (pre-treatment) impairment, suggesting, as reported previously for interventions in sv-PPA (McConathey et al., 2017) but at difference with AD (Ahmed et al., 2012; Coppi et al., 2016), that the most clinically severe cases are the most likely to improve their sympthoms with tDCS.

#### Summary

Despite promising resultsfrom a single study, indications for non-invasive stimulation in CBS remain to be further tested and developed.

### Progressive Supranuclear Palsy

#### Clinial Features

Progressive Supranuclear Palsy affects relatively young patients often before 65 years (Golbe, 1994). Its most characteristic clinical features are postural instability, axial and limb rigidity and impairment of vertical eye saccades (Maher and Lees, 1986; Litvan et al., 2003). PSP also comprises alterations in several non-motor cognitive domains, such as visual attention, information processing, memory, executive function and language (e.g., Grafman et al., 1995; Brown et al., 2010; Daniele et al., 2013). Brain damage mostly affects the basal ganglia, the midbrain, and the superior cerebellar peduncle (Kato et al., 2003; Paviour et al., 2006; Looi et al., 2011), as well as prefrontal cortical regions (Cordato et al., 2000, 2005; Paviour et al., 2006) (Figure 3E).

#### TMS and tDCS Studies

To date only three studies have been carried out to rehabilitate cognitive dysfunction via NIBS. A pre-clinical study evaluated the effects of left anodal DLPFC tDCS and right cathodal DLPFC tDCS, compared to sham stimulation on language impairments. Stimulation enabled significant improvements in a category judgment task with cathodal tDCS and a verbal fluency task with anodal tDCS (Valero-Cabré et al., 2019). Another recent study (Madden et al., 2019) reported the effects of anodal tDCS over the left DLPFC on the language impairment of a single PSP patient. The patient received stimulation concomitantly with language tasks (verbal fluency, naming, reading, and conneceted speech) and, during stimulation, performance in phonemic fluency and action naming tasks improved. Finally, a study by Alexoudi et al. (2019) applied anodal tDCS over the motor and pre-motor cortex and reported that after 10 stimulation sessions, patients increased their visuo-motor co-ordination and processing speed, auditory-verbal learning, episodic memory and phonological fluency.

#### Summary

In view of rTMS or tDCS preliminary evidencecoming from other neurodegenerative diseases and from only two tDCS studies, these techniques may have the potential to improve motor symptoms and also language impairments in PSP patients. Nonetheless further studies using periodical stimulation regimes combined with behavioral and physiological measures are now necessary to confirm such promise.

### Posterior Cortical Atrophy

#### Clinical Features

Posterior Cortical Atrophy impacts relatively young patients in their mid-50s or early 60s. Its most frequent deficits are visuospatial and visuo-perceptual impairments (Renner et al., 2004; Kas et al., 2011; Crutch et al., 2012), and it is often associated with AD pathology (Montembeault et al., 2018). Anatomically, the most affected regions are the parieto-occipital regions and the caudal portions of the temporal lobe, with a right-side predominance (Migliaccio et al., 2009; Lehmann et al., 2011; Figure 3F).

#### TMS and tDCS Studies

Only one study explored the effects of NIBS in a single case of PCA (Gramegna et al., 2018). After 3 months of cognitive treatment followed by two cycles of 1 month of periodical tDCS sessions on the left DLPFC, the patient showed improvements in immediate visual memory, visual global perception, visual attention, and visuo-spatial short-term memory (Gramegna et al., 2018). Importantly, the authors used resting state fMRI and showed after two therapeutic tDCS cycles bilateral increases of DLPFC activity and decreases in the default mode network, particularly for the medial PFC and the precuneus.

#### Summary

Patients with PCA are rare, hence hard to study in large numbers. However, the early age of onset, the atrophy impacting focally parietal and occipital lobes, their spatially-specific gradient of clinical progression, and the rich evidence on rTMS/tDCS effects in perception and spatial attention, make them suited for NIBS interventions.

### General Conclusion

The current review of NIBS studies shows that rTMS and tDCS have been evaluated quite extensively for the improvement of cognitive impairments in neurodegenerative diseases. Yet the large variety of stimulation strategies, parameters and patterns used to evaluate efficacy, the diversity of cognitive tasks, and the scarcity of adequately controlled double-blind sham-controlled studies in homogenous patient populations preclude at this time a reliable meta-analysis of therapeutic applications.

## Information-Based Neurostimulation Strategies to Improve NIBS Efficacy

The field of NIBS research has shown during the last decade an outstanding degree of dynamism and innovation. Basis science, pre-therapeutic and therapeutic TMS/tDCS studies have expanded our knowledge on how NIBS may operate, and opened new avenues for the characterization and development of cognitive rehabilitation treatments in neurology and psychiatry. Nonetheless, an effective use of NIBS requires the neurostimulation community to move beyond classical approaches and fully integrate growing neurophysiological and neuroanatomical evidence subtending cognitive function and dysfunction.

The notion of *Information-based neurostimulation* (Romei et al., 2016) may prove particularly beneficial in improving the therapeutic outcomes of NIBS in neurodegenerative diseases. This framework puts forward a selection of NIBS strategies and parameters based on a detailed characterization of brain activity patterns (ongoing or task-evoked), critical for encoding cognition, considering their state and changes along the course of a disease (Gutchess, 2014). More specifically, NIBS technologies best suited for achieving specific states of brain activity facilitating cognition (Romei et al., 2016) are the ones to be identified and then evaluated therapeutically. Within this framework, we would like to complete the current comprehensive review by briefly presenting six active domains of NIBS research, which offer interesting avenues for therapeutic innovation and optimization to better understand neurodegenerative diseases.

### Network Spread and Functional Connectivity Impact of TMS/tDCS

**FIGURE 7:**
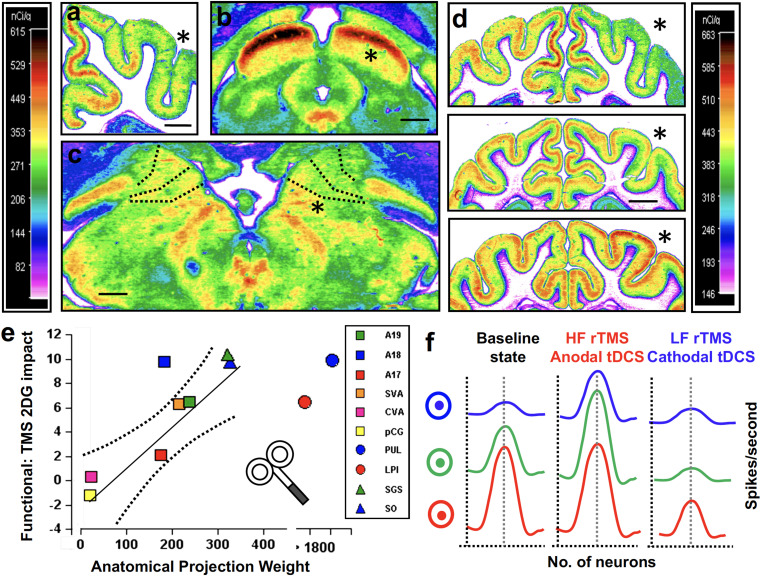
Effects of rTMS and tCS stimulation are pattern- and time-dependent, and their local effects distributed across networks **(a–e)** and influenced by the level of activity on targeted regions and associated networks **(f)**. These should be considered when planning therapies for neurodegenerative diseases, progressing along specific anatomical networks. **(a–e)** rTMS in animals provided high spatial resolution evidence of network specific effects in stimulation. Felines unilaterally stimulated on the posterior parietal cortex (PPC) were injected with a marker of metabolic activity (^14^[C]2DG). Analyses revealed local PPC effects, which proved frequency-dependent **(d)**, showing suppression during high frequency patterns **(top)**, and mild suppression **(middle)**, or enhancements **(bottom)** of local activity outlasting low and high frequency rTMS, respectively. Importantly, distant cortical impacts (visual areas A17, A18, A19, SVA, CVA, pCG) were present in panel **(a)**, the midbrain (superficial layers of the superior colliculus SGS, SO) and **(b)** the dorsal thalamic nuclei (Pulvinar or Postero-lateral; PUL, LPl) **(c)**, all richly connected with the targeted PPC. **(e)** rTMS impact on such regions correlated positively with the density of their structural connections with the targted PPC according to tracing techniques (see Valero-Cabré et al., 2005, 2007). **(f)** The state-dependency nature of TMS modulation has been demonstrated in the visual domain by means of feature selective stimulation or adaptation paradigms, manipulating the activity of specific neuronal subpopulations. Schematic model showing the impact of excitatory or inhibitory stimulation (e.g., high/low frequency rTMS. iTBS/cTBS or anodal/cathodal tDCS) on three populations of sensory neurons (in blue, green, and red) with different levels of baseline activity (depicted as firing rate distribution). Neurons at a low state of activity (blue) will be responsive to excitatory stimulation but eventually refractory to inhibitory TMS/tDCS stimulation patterns.Vice-versa, neurons at a high state of activity (red) will be weakly responsive to excitation but intensively suppressed by inhibitory TMS/tDCS stimulation patterns. Finally, neurons at an intermediate activity level are the most likely to be modulated either excitatorily or inhibitorely. By “priming” their tuning curves (either suppressing via adaptation or enhancing via activation paradigms), state dependence properties may allow TMS/tDCS stimulation to achieve higher modulatory magnitude and more focal and specific effects acting on specific subsets of neurons.

Evidence provided by PET (Paus et al., 1997, 2001a; Chouinard et al., 2003), MRI (Ruff et al., 2006; Bestmann et al., 2008; Polania et al., 2012; Pereira et al., 2013; Kunze et al., 2016), EEG (Ilmoniemi et al., 1997; Taylor et al., 2007), EMG (Valero-Cabré et al., 2001) in humans, and 2-deoxyglucose-PET (Valero-Cabré et al., 2005, 2007; Wagner et al., 2007b) in animals have demonstrated that NIBS combine a local impact on transcranially targeted cortical regions with modulations of functional connectivity across extended brain networks (Figures 7a–d). Network effects are strongly influenced by the richness of white matter connectivity linking the stimulated cortical targets with other brain regions (De Lucia et al., 2007; Quentin et al., 2013, 2015, 2016; Figure 7e).

**FIGURE 8:**
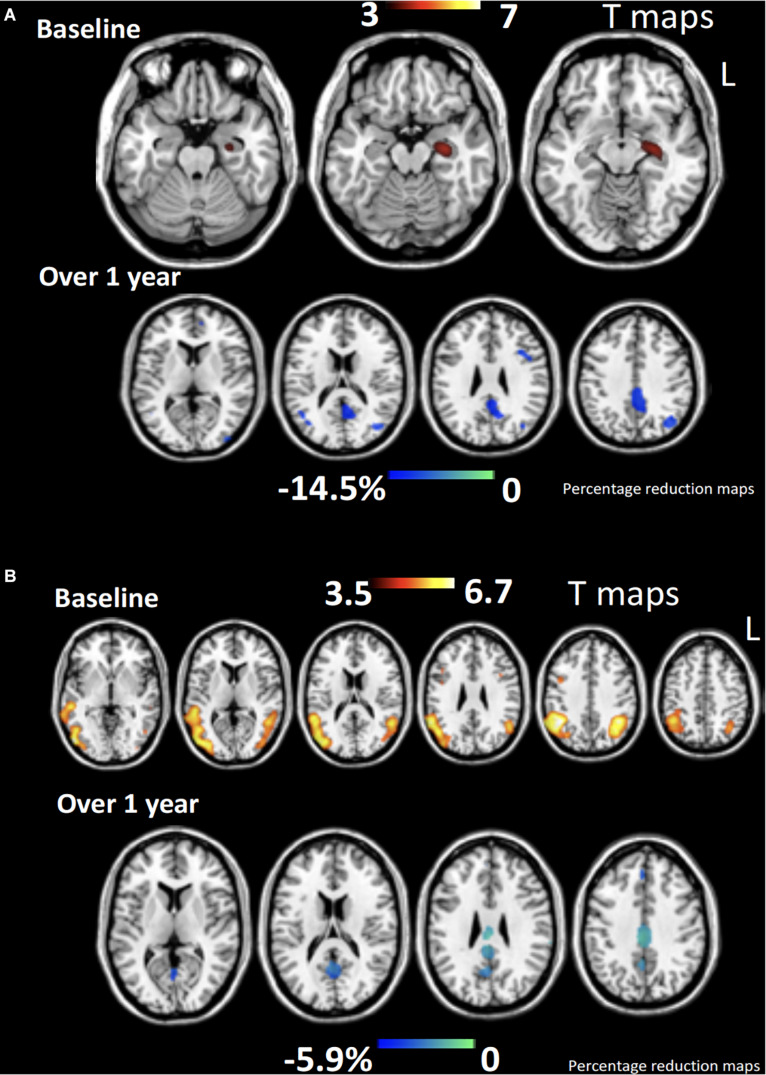
Voxel based morphometry study. Patterns of gaey matter atrophy for late onset Alzheimer’s disease (AD) **(A)** and early onset AD **(B)** at the time of diagnosis (baseline) and after 12 months progression, compared to healthy controls. Color bars denote T values **(top)** and percentage of GM reduction during follow up **(bottom)**. Note that whereas late onset AD patients (>65 years) show signs of atrophy limited to the medial temporal regions, early onset AD patients (<65 years) suffer widespread atrophy in temporal, parietal, occipital, and frontal cortices. One year thereafter, atrophy progression for both early and late AD onset patients impacts the same regions of the default-mode network (Adapted with permission from the copyright holders from Migliaccio et al., 2015).

This is particularly relevant for neurodegenerative diseases, because these progress through specific functional (Seeley et al., 2009; Farb et al., 2013; Guo et al., 2013; Liu et al., 2014) and white matter structural (Clavaguera et al., 2013; Maruyama et al., 2013; Pievani et al., 2014) networks, a process that can be paralleled by the network specific impact reported for NIBS. Importantly, neurodegeneration generally starts long time before the onset of clinical manifestations, during the so-called “asymptomatic” stage (Hampel et al., 2010). The subtending pathological mechanisms impact specific sets of regions and the distribution of anatomical damage evolves dynamically from early to later stages depending on the level of damage spread (Przedborski et al., 2003). Illustrating this point, a recent study (Migliaccio et al., 2015) reported that whereas late onset AD patients (>65 years) show signs of atrophy limited to the medial temporal regions, early onset AD patients (<65 years) suffer widespread atrophy in temporal, parietal, occipital and frontal cortices, sometimes long before they reach 65 years old (Figure 8). Interestingly however, after one year, patterns of atrophy progression for both early and late AD onset patients impact the same regions of the so-called default-network (Migliaccio et al., 2015; Figure 8), which is particularly vulnerable to AD pathology.

**FIGURE 9:**
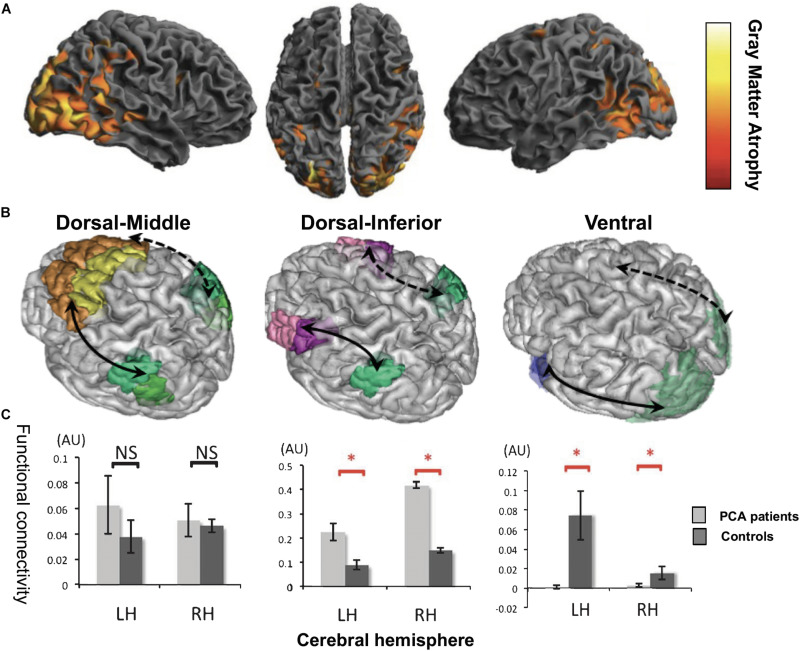
Voxel based morphometry and fMRI studies. **(A)** Regions of gray matter atrophy in posterior cortical atrophy (PCA) patients, compared to healthy controls. The color scale indicates the level of atrophy (yellow signaling highe levels). Cortical thinning in posterior parietal and occipital regions characterizes PCA patients. **(B)** A representative brain depicting the seed regions employed to estimate levels of functional connectivity (FC) including the dorsal-middle **(left)** and dorsal-inferior **(middle)** components of visual networks, and also the ventral visual network **(right)**, in which PCA patients show abnormal functional connectivity, compared to healthy controls. Arrows depict measures of FC between seed regions. Continuous and dotted arrow lines indicate intra-hemispheric left and right interactions, respectively. **(C)** Histograms quantifying FC levels for right (RH) or left (LH) intra hemispheric interactions in PCA patients (light gray columns) vs healthy controls (dark gray) (AU, Arbitrary Units ^∗^, corrected *p* < 0.05, NS, non-significant). PCA patients show a trend towards FC increases for the dorsal-middle visual network **(left)**, significant FC increases for the dorsal-inferior visual network **(middle)**, however, significant FC decreases for the ventral network **(right)** (Adapted with permission from the copyright holders from Migliaccio et al., 2016).

To fully understand the potential of NIBS on cognitive symptoms of neurodegenerative diseases, some recent key advances need to be summarized. Under a purely topological point of view, for many decades, neurodegenerative diseases have been conceived as impacting focal brain regions, and progressing nonspecifically across brain areas hence, not following any specific spatial pattern. The advent of advanced brain imaging techniques has shown, however, that their spatial spread impacts brain sites organized in networks (Seeley et al., 2009) by following specific white matter pathways (Clavaguera et al., 2013; Maruyama et al., 2013; Pievani et al., 2014). Accordingly, damage translates into sets of symptoms, in coherence with the anatomical damage reached at each clinical stage of the disease (Saxena and Caroni, 2011). According to the *network degeneration hypothesis*, neurodegenerative diseases can be conceptualized as connectivity disorders (Seeley et al., 2009) originating in small focal networks to progressively spread to interconnected areas (Gomez-Ramirez and Wu, 2014). Furthermore, disease-specific areas of vulnerability can be tracked with PET imaging using radioactive ligands, binding specific proteins expressed by cells in distress (Morrison et al., 1998) or binding specific pathologic aggregates (as tau proteins) (Saint-Aubert et al., 2017). Nonetheless, the underlying mechanisms of such a network distribution process remain poorly understood (Gomez-Ramirez and Wu, 2014). Among other hypotheses, anatomical progression could be explained by spatially-specific patterns of network vulnerability, i.e., neurochemical fragility of neuronal populations (such as those of Von Economo fronto-insular neurons in bv-FTD, Seeley et al., 2009), sensitive to stressors, and combined with covariates such as genetic background, age or preexisting conditions (e.g., misfolded protein-related disorders) (Saxena and Caroni, 2011). Recent reports have also supported processes of cell-to-cell transmission of misfolded proteins (Clavaguera et al., 2013; Herva and Spillantini, 2015), by aggregates transported across neurons reaching specific sites, able to transfer across synapses. These biological mechanisms will be ultimately responsible for the disease-specific breakdown of functional connectivity that can be revealed by alterations in the normal patterns of resting state fMRI in patient populations, opening new avenues for the development of biomarkers and predictors of clinical and anatomical prognosis. For example, a recent study in PCA patients (Migliaccio et al., 2016) has shown reduced functional connectivity in the ventral cortical visual network, whereas in contrast, functional connectivity was found increased in the inferior component of the dorsal visual network (Figure 9). Moreover, greater GM atrophy in occipital regions, which are typically impacted in these patients, correlated with increased functional connectivity (Migliaccio et al., 2016).

Combining whole-brain high spatial resolution imaging methods with tasks, the impact of brain stimulation in the injured brain becomes not only therapeutically relevant but also highly informative about the mechanisms by which local plasticity, neural reorganization and/or function remapping could potentially allow recovery. For example, sv-PPA patients suffer consistent decreases of functional connectivity between the anterior temporal cortices and a broad range of other brain regions (Guo et al., 2013). Interestingly, the delivery of tDCS over the left or right ATL in these patients entrained significant improvements in semantic processing (Teichmann et al., 2016), suggesting that structural and functional connectivity between these regions is critical for enabling semantic processes. The combined use of neuroimaging techniques prior and following stimulation could help tease out which network seeded in the ATL is necessarily recruited in semantic processing (Gutchess, 2014). Moreover, contrasting activity changes across different cortical regions to enhance specific cognitive functions in patients can potentially shed light on the most important networks and regions contributing to that function, helping to develop more efficient evidence-based treatments to tackle with this pathology.

### State-Dependent Effects of TMS and tDCS Stimulation on Brain Systems

Another influential finding in the NIBS field is that the direction and magnitude of the modulatory effects is strongly influenced by ongoing activity operating on the targeted region at the time of stimulation (Silvanto and Muggleton, 2008; Silvanto and Pascual-Leone, 2008; Silvanto et al., 2008; for a review). To this regard, it has been shown that when a TMS/tDCS pattern targets a cortical region hosting neuronal subpopulations processing different functions or features, this tends to differentially influence neuronal resources as a function of their ongoing excitability levels. In the visual system for example, excitatory rTMS patterns are more likely to induce further lasting excitation on neurons kept at a low level of excitability. In contrast, inhibitory rTMS patterns would induce further suppression of neuronal clusters kept at a high level of excitability (Silvanto et al., 2007; Figure 7f).

Based on the state-dependency principles, some TMS/tDCS studies aiming to improve cognition in neurodegenerative conditions have applied stimulation concurrently with online cognitive tasks in AD patients (Bentwich et al., 2011; Brem et al., 2013; Rabey et al., 2013; Cotelli et al., 2014a; Lee et al., 2016; Rabey and Dobronevski, 2016) and PPA patients (Cotelli et al., 2014b, 2016; Tsapkini et al., 2014; Gervits et al., 2016; McConathey et al., 2017; Roncero et al., 2017). A majority of these reports showed significant cognitive improvements compared to pre-stimulation performance (Bentwich et al., 2011; Brem et al., 2013; Rabey et al., 2013; Cotelli et al., 2014b, 2016; Tsapkini et al., 2014; Gervits et al., 2016; Lee et al., 2016; Rabey and Dobronevski, 2016; McConathey et al., 2017). Nonetheless, none of these studies compared the former with an active or sham stimulation condition without the use of that sametask, a control that would be necessary to highlight the benefits of task-stimulation coupling and demonstrate priming effects on the activity of the targeted region. Generally speaking, the interactions between tasks activating the targeted systems and transcranial stimulation may prove complex and not necessarily synergistic. For these reasons, in case of doubt – unless a previously tested facilitatory paradigm can be employed – it is recommended to implement a simple task, ensuring that all patients will remain in a similar brain state during stimulation.

Nonetheless, behavioral approaches such as task adaptation (i.e., sustained exposure to an invariant sensory pattern which decreases neuronal activity of the neuronal population processing a specific feature) could be employed prior, during or even following TMS/tDCS to maximize the effects driven by excitatory stimulation patterns. Similarly, sensory, motor or cognitive priming tasks shall be applied to increase ongoing levels of activity, during or even following stimulation to facilitate the suppressive effect of inhibitory TMS/tDCS patterns and render such populations less sensitive to stimulation (e.g., see example in visuospatial attention in Chanes et al., 2012). In neurodegenerative patients, one of the main sources of brain state modulations which needs to be accounted for is the impact and progression over time of cortical damage and atrophy. Therapeutically, a well-suited manipulation of the activity state may allow clinicians to shape the direction, selectivity and magnitude of the neurostimulatory effects on regions hosting mixed neuronal populations with a diversity of functions, and by doing so overcome limitations in TMS/tDCS spatial resolution, and maxime cognitive impact.

### Manipulation of Oscillatory Activity and Interregional Synchrony Relevant for Cognition

**FIGURE 10:**
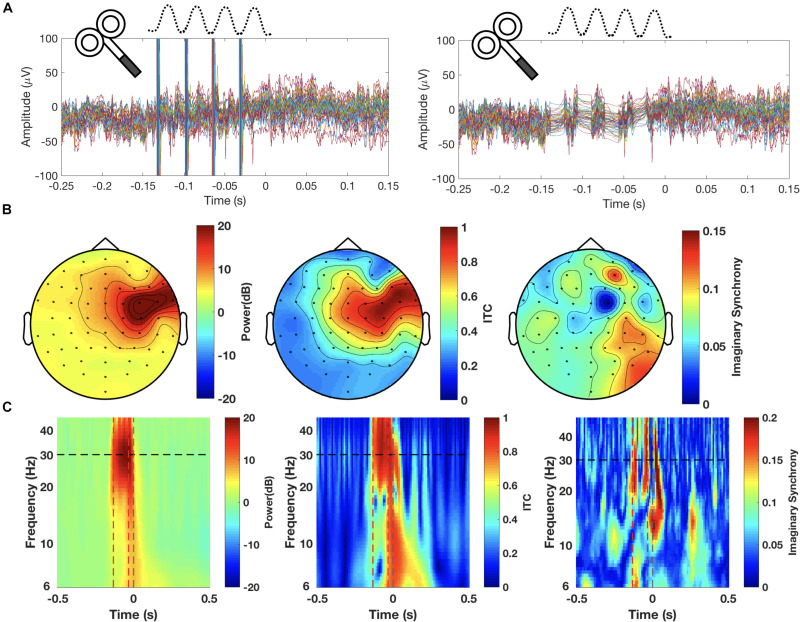
Rhythmic TMS or fluctuating tCS (aka tACS) have been used to explore the frequency specific modulation of oscillations and interregional synchrony of brain systems. Rhythmic modulations **(A–C)** on a representative healthy participant, stimulated with brief four TMS pulses at 30 Hz (high-beta frequency) under EEG monitoring delivered to a right frontal region (FEF, Frontal Eye Fields), during a visual detection task. **(A)** Raw EEG recordings (64 electrode scalp array) prior **(left)** and following **(right)** TMS artifact removal and signal interpolation procedures. Topographies (25–35 Hz band) during delivery of TMS **(B)** and time frequency analyses on electrode FC2, closest to the FEF **(C)** are shown for power **(left)**, inter-trial coherence [ITC, **(middle)**], and fronto-parietal synchrony **(right)**. About 30 Hz TMS bursts (100 ms total duration) increased power and align the phases of high beta oscillators on the right FEF. They also increased level of synchrony between the right FEF and parietal regions at this same frequency band. Dotted white circles indicate the position of the FC2 EEG electrode (closest to the stimulated FEF). Horizontal black dotted lines signal the frequency of the delivered rTMS bursts. Vertical red dotted lines indicate the time of the 1st and 4th pulse of each rTMS bursts and the vertical gray line signal the time of the visual target onset.

The third and most recent finding influencing the use of NIBS is the ability of frequency-specific rhythmic TMS bursts or sinusoidal tDCS patterns to either enhance and/or impose oscillatory activity involving cyclic fluctuations of activity in local clusters of cortical neurons (see Thut and Miniussi, 2009; Gross et al., 2011; Figure 10). Additionally, locally entrained rhythms can also be conveyed to distant regions, enhancing temporally correlated activity or interregional synchrony. Research in this area has also shown that single TMS pulses have the ability to phase-reset and align local oscillators in a given cortical region and boost transiently the amplitude of rhythms at the so called “natural frequency” that such oscillators are most likely to generate (see pioneering evidence by Paus et al., 2001b; Rosanova et al., 2009).

**FIGURE 11:**
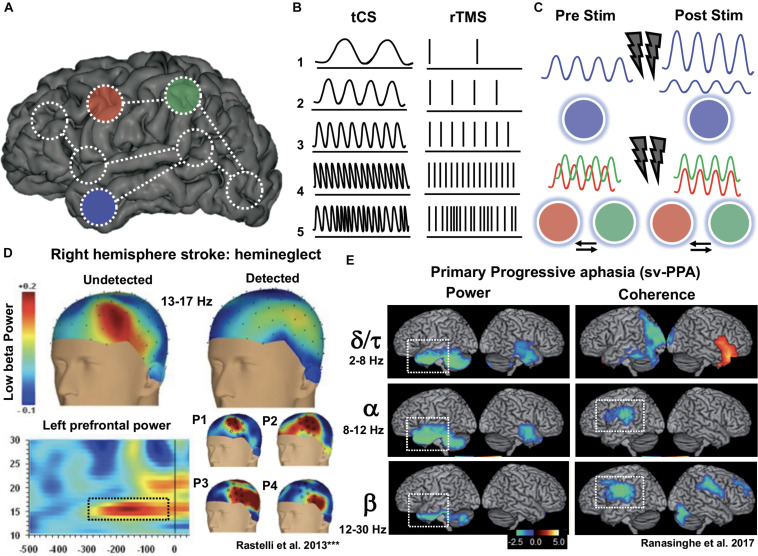
Neurophysiological techniques such as scalp EEG or MEG have characterized pathological brain states as alterations of local or interregional oscillatory activity. In a given brain **(A)** the latter could be shown as a frequency-specific bands dysfunction of power, amplitude or coherence at (e.g., *blue* anterior temporal pole ROI) or as pathological levels of synchronization between regions within a network (e.g., *red-green* fronto-parietal ROIs). **(B)** Transcranial current stimulation (tCS) and rTMS generate electrical or magnetic rythmic fields, respectively, able to entrain neurons; Additionally **(E)**, non-frequency specific tCS fields or irregular jittered random rTMS bursts (electrical noise) may serve to desynchronize ongoing rhythmic activity or prevent the build-up of rhythmic activity **(C)**. To improve the deficits subtended by *oscillopathies* **(C, top)** fluctuating/rhythmic tCS/rTMS could be delivered to modulate oscillatory activity. In contrast, irregular or random stimulation may lead to opposite effects. Similarly, **(C, bottom)** tCS/rTMS patterns delivered on a single network location could modulate synchronization between interconnected areas. Potential clinical applications of such principles are being piloted in stroke patients **(D)** suffering hemispatial neglect; MEG studies have identified a build up of left prefrontal low-beta (13–17 Hz) synchrony prior (-250 ms) to the onset of visual targets patients neglect to acknowledge (modified from Rastelli et al., 2013 with permission). In such case, jittered rTMS or tRNS patterns could be applied to desynchronize maladaptive left prefrontal low-beta activity associated to this syndrom. In the same vein **(E)**, a recent MEG study characterized *oscillopathies* in sv-PPA patients and showed broadband power and/or synchrony deficits (δ/τ, α, β bands) in left and right temporal, posterior parietal, temporoparietal, and inferior frontal regions. Most discriminative MEG findings (not influenced by temporal cortical atrophy) consisted in an alpha and beta hypo-synchrony in areas of the posterior language network. Frequency-tailored oscillated tCS (e.g., alpha and beta tACS, on left temporal or inferior parietal areas) could be tested to improve language deficits in sv-PPA patients. [**(D,E)** were adapted with permission from authors and the copyright holders, from Rastelli et al., 2013 and Ranasinghe et al., 2017].

These effects are relevant because cortical oscillations are now considered as an essential mechanism underlying specific cognitive operations and behaviors (Buzsáki and Draguhn, 2004). Accordingly, local and/or interregional entrainment of rhythmic TMS patterns have been shown to facilitate cognitive processes. For example, relevant for neurodegenerative diseases, the lasting *echo* of rhythmic beta TMS patterns (beyond burst duration) has shown to induce impairments of memory consolidation in inferior prefrontal frontal regions (Hanslmayr et al., 2014). Likewise, online high-beta (30 Hz) TMS bursts to the left FEF prior to the onset of a near threshold visual stimulus degraded visual perception whereas equivalent TMS random patterns enhanced it (Chanes et al., 2015). These results suggest that the effects of rhythmic entrainment on cognition could be potentially relevant for modulating behavior in the healthy but also for treating neurological conditions. Moreover, effects proved in any case site-, phase- and frequency-dependent, and showed that for some cerebral regions and cognitive processes (e.g., attention), it is the desynchronization of rhythmic activity that can also result in cognitive enhancements (Thut et al., 2017). Similarly, tACS has also suggested an ability to entrain cyclic activity (Helfrich F. et al., 2014; Helfrich R. et al., 2014; Herrmann et al., 2016) and when delivered occipitally at 10 Hz, for example, oscillated current co-cycles with alpha occipital activity and facilitates phase-dependent visual perception (Thut et al., 2011). Moreover, tACS makes possible the use of complex patterns based on “nested” high-frequencies (gamma band) on top of slower underlying theta rhythms, which delivered pre-frontally have shown recently to enhance working memory capacity (Alekseichuk et al., 2016; Figures 11A–C).

Alterations of oscillatory brain activity and neural synchrony emerged some years ago as novel potential biomarkers of cognitive neurological deficits following focal stroke lesions such as visuospatial neglect (Rastelli et al., 2013; Yordanova et al., 2017; Figure 11D). Similarly, such alterations are also being reported for neurodegenerative diseases (see Vecchio et al., 2013; Meder and Siebner, 2018; for reviews). Although mechanistic causes and consequences remain unclear, AD patients exhibit for example relative power increases for slow oscillations (delta and theta rhythms) and in contrast decreases of fast rhythms (alpha, beta, and gamma rhythms) (see Vecchio et al., 2013; Hamm et al., 2015; for a review). Alterations of oscillatory activity and local or interregional synchrony have also been found more recently in other neurodegenerative diseases (Andersson et al., 2008; Ponsen et al., 2013; Ranasinghe et al., 2017). A MEG study comparing PD patients with or without dementia found differences in oscillatory power between these two groups, the former showing lower power in the alpha and beta bands in occipito-parieto-temporal and frontal areas compared to the latter, but stronger activation in the delta and theta bands in parieto-occipital and fronto-parietal areas (Ponsen et al., 2013). Patients with DLB have also displayed increased delta and theta activity and decreases of alpha and beta rhythms compared to healthy participants and to AD patients (Andersson et al., 2008). Most interestingly, Ranasinghe et al. (2017) reported MEG evidence of dysfunctional patterns of alpha and beta neural synchronization in PPA patients. Recordings revealed PPA-variant specific patterns of hypo- and hyper-synchrony. These alterations remained significant even after correcting for gray matter volume, hence supporting the idea that such alterations (known as oscillopathies) reflect genuine functional alterations of neural activity, and cannot be solely explained by cortical atrophy (Ranasinghe et al., 2017; see Figure 11E).

In a similar vein a recent MEG study assessed, in five different neurodegenerative conditions, direct coherence measures and calculated nodal local efficiency, a proxy of how well connected a node is with its network neighbors, hence how resilient can be to neural damage. Using a data driven whole-brain connectivity analytic approach on resting state MEG data, authors successfully searched for characteristic neurophysiological signatures, likely distinctive in spatial and frequency profiles for AD, PCA, bv-FTD, PSP, and nfv-PPA patients. The study was able to cluster clinical syndromes sharing a similar underlying network pathology (referred to as “circuitopathy”) and reported for example decreases in network efficiency in the gamma band for AD and PCA, whereas alterations in bv-FTD, PSP and nfv-PPA impacted lower frequencies (delta, alpha, and low gamma) (Sami et al., 2018).

Finally, a very recent study in bv-FTD used a hypothesis driven single network analyses of MEG data during the generation and inhibition of responses using a Go-NoGo motor task and explored alterations of cross-frequency coupling phenomena (Hughes et al., 2018). Authors concluded a reduction of event related beta-band desynchronization – scaling with behavioral des-inhibition – and also deficient beta rebound re-synchronization. Further analyses revealed also a general reduction of within and cross-frequency coupling between three regions key for inhibitory control such as the IFG, the pre-supplementary motor area (pre-SMA) and the primary motor cortex (M1; Hughes et al., 2018). As the former study, this report emphasizes the notion of network- and band- specific alterations of oscillatory activity caused by cortical damage and/or the ensuing functional reorganization, and their role subtending the behavioral phenotypes of neurodegenerative diseases (Hughes et al., 2018; Meder and Siebner, 2018; Sami et al., 2018).

In sum, electrophysiological evidence suggests a disorganization of functional circuits and alterations of neural synchrony at early stages of neurodegenerative diseases preceding structural atrophy changes (Jagust, 2013; McBride et al., 2014; Ahnaou et al., 2017; Bonakdarpour et al., 2017). Moreover, spatio-temporal synchrony abnormalities reflect a breakdown of cytoarchitectural network properties and/or their struggle to compensate damage, hence account for brain resilience. Most important, spatio-temporal correlates of network dysfunction have a bearing on symptoms suffered by patients, hence can be used to tease apart disease variants (Ranasinghe et al., 2017; Sami et al., 2018) or specific neurodegenerative phenotypes (Hughes et al., 2018; Sami et al., 2018). The ability of some NIBS techniques, notably rhythmic TMS and tACS, to modulate oscillatory activity and interregional synchrony, will provide new opportunities to intervene on specific neurodegenerative diseases, with the aim to re-instate oscillatory normality across altered networks and in turn slow-down the progression of cognitive decline (Figure 11).

The use of oscillation-based rhythmic neuromodulation principles in the rehabilitation of cognitive deficits in neurodegenerative patients remains to be developed. Nonetheless, successful application of anti-phasic tACS individually tailored for tremor in PD patients may show an interesting path to follow to improve cognitive impairments (Brittain et al., 2013). Indeed, as the role of local and widespread oscillatory/synchrony activity in cognitive coding is being associated to many aspects of high-level cognition, the development of rhythmic stimulation is emerging as a promising therapeutic domain.

### Computational Models of Current Distribution to Customized NIBS Dosing

A myriad of variables (e.g., scalp-to-brain distance, skull shape, cortical thickness, sulcal pattern and gyral geometry, relative depth and orientation of neuronal layers and local excitability thresholds, etc.) will determine effective current intensity received by a brain region. In absence of direct physiological evidence, finite element methods (FEM) biophyisical computational models can be employed to estimate the spread and intensity of current fields based on individual structural MRI, and individually tailor stimulation settings (Figure 6). Such models consider cortical anatomical anisotropies (e.g., sulci, gyri, atrophy, lesions, etc.), biophysical properties (permittivity/conductivity) and the volume of tissue layers that TMS/tDCS fields need to cross before reaching a cortical target. On such basis, they estimate the cortical site of peak current and the radial spatial distribution of the electrical fields. Off-the-shelf computational models for TMS and tDCS (simNIBS 2.0,^2^ Thielscher et al., 2015; or ROAST,^3^ Huang et al., 2017) are now freely available for users to plan stimulation settings.

### Toward New Families of Brain Stimulation Technologies

The field of NIBS technologies is continuously developing novel stimulation technologies to be used in human clinical settings. In an attempt to overcome some of the limitations of TMS and tDCS, particularly noteworthy has been the search for devices capable of inducing electrical fields of higher magnitude, in a more focal and steerable manner, and when possible directly into subcortical brain structures without having to influence all layers of non-neural and neural tissue before reaching a cortical target.

Some of such novel technologies have moved away from electromagnetic sources and embraced transcranially-delivered mechanical energy *via* focused ultrasound (FUS) sources (Tufail et al., 2010; see Tyler et al., 2018 for a recent review). Others have developed new uses of transcranial Electrical currents (tECS) by using extremely short pulses of direct electrical current with a rotating electrical gradient between an array of multiple pairs of tDCS electrodes, converging on a single cortical location. This approach, termed intersectional short pulse (ISP) stimulation taking advantage of a slow temporal summation in neuronal bodies, allows the injection of high currents into a brain location (>0.7–1 mV/mm), while keeping charge density low and scalp skin sensations bearable (Vöröslakos et al., 2018). Finally, Grossman et al. (2017) have recently reported in rodents the ability to generate a deep, focal, and steerable deep temporal interference stimulation (TIS). Effects are generated by merging within a superficial or deep spatial gradient around a brain target two high-frequency oscillating transcranial electrical fields (equivalent to tACS) slightly shifted in frequency (Grossman et al., 2017).

All in all, current research and therapeutic applications of neuromodulation in humans keep relaying in rTMS and tDCS/tACS approaches. There is however little doubt that alternative currently developing techniques (such as FUS, IPS, or TIS) initially explored in rodent models eventually will be transferred to human patients with the ambition to expand the array of neuromodulation technologies at the service of neurology (Krishna et al., 2018; Kubanek, 2018; see Grossmann, 2018 for reviews).

### Toward New “Patients” and New Ways to Apply NIBS

The symptomatology of neurodegenerative diseases appears many years after the onset of brain damage, once this is already very widespread. Recent failures of potential disease-modifying drugs, in particular for AD, most probably reflect the fact that patients enrolled in clinical trials are pathologically far too advanced for therapies to be able to derive meaningful clinical benefit. Whith this in mind, it is clear that a major aim for clinical research in this field is to identify patients long beforethey develop symptoms.

The single or combinatorial use of different biomarkers has demonstrated a high potential to diagnose and track the progression of neurodegenerative diseases. However, the identification of biomarkers for preclinical diease detection is essential to make crucial therapeutic progress. For example, several biomarkers are available for AD: positive amyloid or tau tracer retention on PET imaging; low cerebrospinal fluid (CSF) concentrations of the amyloid-β 1-42 peptide, high CSF concentrations in total tau and phospho-tau; mesial temporal lobe atrophy on MRI, and/or temporoparietal/precuneus hypometabolism or hypoperfusion on PET (for a review see Weiner et al., 2015). Many of these biomarkers have also been tested on cohorts of normal individuals to detect early signs of AD (Weiner et al., 2015). The main goals are: on one hand to identify new easy-to-test biomarkers (e.g., plasma or retinal) as early as possible, and, on the other hand, to make progress in the early identification of individuals at “high risk” for the disease rather than of early phases of the disease. For the most simple cases, in which the risk of dementia is genetically determined (e.g., C9orf72 mutation eading to bv-FTD), early diagnosis is feasible at pre-clinical stages of the disease (De Jesus-Hernandez et al., 2011; Balendra and Isaacs, 2018). For other clinical cases, it it is important to identify potential high-risk factors; as for example, becoming old and being a homozygous carrier of the Apolipoprotein E4 allele, which increases the risk of AD (Liu et al., 2013). Within this framework, NIBS will be most clinically effective if it can be applied at very early stages, on very specific brain locations and systems before damage disseminates widspreadly. Potentially interesting targets to tap on at such early stages are the neurotransmitter systems. For example, cognitive functions such as verbal learning, memory, attention, and working memory are glutamate dependent (e.g., Rowland et al., 2005), and this same neurochemicals systems can be cortically activated by the after-effects of several NIBS protocols(e.g., Huang et al., 2007). Likewise, more recently, findings support a causal role for the dopamine precursor L-tyrosine in mediating the effects of NIBS on verbal working memory (Jongkees et al., 2017). In older AD patients, rTMS facilitates cortical plasticity via administration of rotigotine, a dopamine agonist (Koch et al., 2014). This evidence strongly suggests that some of the effects of NIBS can be induced by the modulation of specific neurotransmitters. In sum, since most psychotropic drugs act at the level of neurotransmitters, future strategies should aim to improve cognition by using drugs targeting brain neurochemical, systems whose specific action could be further enhanced by an association with NIBS approaches.

## Final Remarks and Conclusion

In a context characterized by increasing population aging and the lack of effective treatments for age-associated neurological conditions, the search for novel therapeutic approaches beyond pharmacology and cognitive rehabilitation is gaining momentum. The evidence reviewed in this paper suggests that personalized NIBS-based *electroceuticals* have the ability to drive improvements in memory, attention and language in patients with neurodegenerative diseases. Yet, the real value of such approaches, mainly tested in AD and PPA, remains to be consistently evaluated and verified using larger homogeneous cohorts of well-characterized patients, more adequate designs and notably double-blind sham-controlled trials, and sensitive cognitive assessments in combination with neuroimaging and neurophysiological evidence. To this end, determining the NIBS settings, strategies and parameters most likely to result in effective therapeutically meaningful outcomes and the use of comparable and reproducible evaluation methodology is paramount. Moreover, a further understanding of structural and physiological variables influencing the interaction of TMS and tDCS electrical currents with head/brain tissue layers is also relevant to tailor to each individual patient, target location, stimulation patterns and dosing and to improve computational Finite Element biophysical head-brain-models of current distribution.

However, we here suggest that attaining full potential for therapeutic success will require the consideration of information-based neurostimulation principles put forward recently, i.e., cutting-edge knowledge on anatomical (sites and networks subtending function) and neurophysiological features (i.e., coding patterns subtending cognitive operations) characterizing each specific neurodegenerative condition, and their dynamic changes across disease stages. To such end, it is essential (1) to consider the network-distribution of neurostimulation effects when planning strategies and interpreting outcomes; (2) to monitor and manipulate through specific tasks the level of brain activity prior, during or following stimulation to boost outcomes; (3) to integrate *state-of-the art* knowledge on cognitive coding through local and distributed oscillatory patterns and assess their potential manipulation with NIBS modalities, such as rhythmic-TMS or tACS; and (4) to allow critical and open-minded vigilance needs to be alloted to the emergence of new brain stimulation technologies, which might overcome some of the critical limitations of either TMS or tDCS such as lack of intensity, focality or steerability.

This framework will help build innovative therapeuticrationales on the basis of an accurate characterization of a dynamically evolving anatomical and neurophysiological features (coding, excitability, functional connectivity) of each neurodegenerative disease. Finally, to establish therapeutic value of NIBS and better understand its mechanisms of action it is essential to integrate in studies brain neuroimaging and neurophysiological recordings and use such to monitor brain activity state and characterize network connectivity and their changes with treatment.

## Author Contributions

RM and AV-C were responsible for the coordination of the manuscript. CSa, RM, and AV-C devised the manuscript project and contributed to manuscript redaction. JM, CSt, and JG contributed to the redaction of specific sections of the manuscript. Csa and JG were responsible for the process of literature search. MT contributed to the revision of the manuscript. All authors contributed to the article and approved the submitted version.

## Conflict of Interest

The authors declare that the research was conducted in the absence of any commercial or financial relationships that could be construed as a potential conflict of interest.

## Footnotes

1 https://www.neurology.org/sites/default/files/ifa/loe.pdf

2 https://simnibs.github.io/simnibs/build/html/index.html

3 www.parralab.org/roast/
